# Comparison of the Efficacy of Nonsteroidal Anti-Inflammatory Drugs and Opioids in the Treatment of Acute Renal Colic: A Systematic Review and Meta-Analysis

**DOI:** 10.3389/fphar.2021.728908

**Published:** 2022-01-27

**Authors:** Xie-Yuan Leng, Chang-Ning Liu, Shi-Chan Wang, Hao-Dong Peng, De-Guang Wang, Hai-Feng Pan

**Affiliations:** ^1^ The First Clinical College, Anhui Medical University, Hefei, China; ^2^ Department of Nephrology, Second Affiliated Hospital of Anhui Medical University, Hefei, China; ^3^ Department of Epidemiology and Biostatistics, School of Public Health, Anhui Medical University, Hefei, China; ^4^ Inflammation and Immune Mediated Diseases Laboratory of Anhui Province, Hefei, China

**Keywords:** renal colic, NSAIDs, nonsteroidal drugs, opioids, meta-analysis, systematic review

## Abstract

**Background:** Although multiple randomized controlled trials (RCTs) and systematic review and meta-analysis were performed to investigate the efficiency and safety of nonsteroidal anti-inflammatory drugs (NSAIDs) and opioids in the treatment of acute renal colic, the therapeutic regimen of renal colic is still controversial. Therefore, the aim of this study was to derive a more concise comparison of the effectiveness and safety between NSAIDs and opioids in the treatment for patients with acute renal colic by a systematic review and meta-analysis.

**Design:** We searched PubMed, Embase, and Cochrane Central Register of controlled trials for seeking eligible studies. The pooled mean difference (MD) or risk ratio (RR) with 95% confidence interval (CI) was calculated using the random effects model. The primary outcome was assessed according to the Grading of Recommendations Assessment, Development and Evaluation.

**Results:** A total of 18 studies involving 3,121 participants were included in the systematic review and meta-analysis. No significant difference between the NSAID and opioid groups was observed, with changes in the visual analog scale (VAS) at 0–30 min (MD = 0.79, 95% CI: −0.51, 2.10). NSAIDs in the form of intravenous administration (IV) had no better effect on the changes in the VAS at 0–30 min, when compared to opioids (MD = 1.25, 95% Cl: −4.81, 7.3). The NSAIDs group in the form of IV had no better outcome compared to the opioids group, as well as the VAS at 30 min (MD = −1.18, 95% Cl: −3.82, 1.45; MD = −2.3, 95% Cl: −5.02, 0.42, respectively). Moreover, similar results of this outcome were also seen with the VAS at 45 min (MD = −1.36, 95% Cl: −5.24, 2.52). Besides, there was a statistical difference in the incidence of later rescue (RR = 0.76, 95% CI: 0.66, 0.89), drug-related adverse events (RR = 0.44, 95% CI: 0.27, 0.71), and vomiting (RR = 0.68, 95% CI: 0.49, 0.96).

**Conclusion:** There is no significant difference between the NSAIDs and opioids in the treatment of renal colic in many outcomes (e.g., the VAS over different periods using different injection methods at 30 and 60 min), which has been focused on in this study. However, the patients who were treated using NSAIDs by clinicians can benefit from fewer side effects.

## Introduction

Renal colic, a sort of pain that is hard to bear for patients when it attacks, along with a high incidence of 0.5% each year in Europe and North America, is characterized by pain in the waist or upper abdomen with paroxysmal attack (Patti and Leslie, 2021). Timely pain management is usually performed by using nonsteroidal anti-inflammatory drugs (NSAIDs) and with opioids as a drug of choice for renal colic patients, for reducing acute and unbearable pain in patients who have been severely affected by renal colic (Schmidt and Kroeger, 2016; Zamanian et al., 2016). Nevertheless, the antinociceptive effects of both these drugs are different, as is the pharmacological mechanism. For instance, NSAIDs have been associated with inhibiting the release of prostaglandins, further reducing the pressure of kidneys in order to increase the diuretic effect (Larkin et al., 1999).

However, the application of NSAIDs or opioids depends on the clinical workers' preference to these drugs (Sackner et al., 1974). A study of Passik (2009) indicated that this reluctance to their application may be caused by worries that are relevant to the possible adverse events and other potential risks, including fatal opioid overdose, the development of tolerance and dependence syndrome, and the harmful use of opioid and diversion. Some previous studies have revealed that renal colic patients could get more benefits from applying NSAIDs compared to opioids (Masoumi et al., 2014; Rezaei et al., 2020). Meanwhile, randomized controlled trials (RCTs) have shown a completely opposite conclusion, which hold the view that NSAIDs has a similar analgesic effect to opioids for pain relief in the management of renal colic (Majidi and Derakhshani, 2020).

Compared with a previous meta-analysis on a similar topic, this study has the advantage of including more new RCTs, more abundant outcome indicators, and a detailed collection of analgesic effects of two different drugs at different times in important outcomes, which is believed to play a guiding role in clinical practice.

Moreover, there are a variety of problems about the agents used clinically in the treatment of acute renal colic, that is, the routes of administration [intramuscular administration (IM) and intravenous administration (IV)], dosage of agents, and the safety and effectiveness of agents appropriate on patients with acute renal colic (Marthak et al., 1991). Relevant studies have shown that different routes of administration for pain management could have an influence on the adverse events and others (Fanelli et al., 2014; Daoust et al., 2015). Therefore, based on the uncertainty of pain management of NSAIDs and opioids for renal colic patients, this systematic review and meta-analysis was performed to investigate the efficacy and safety of NSAIDs and opioids in the treatment of renal colic and obtain an optimal management for patients, with less adverse events under the premise of relieving pain.

## Methods

### Search Strategy

Three electronic databases, including PUBMED, Embase, and Cochrane Central Register of controlled trials, were screened up to February 1, 2021, for seeking eligible studies on comparison of efficacy and safety of NSAIDs and opioids in patients undergoing treatment for renal colic. The medical terms and keywords we used are as follows: “renal colic,” “Nonsteroidal drugs,” “non-steroidal anti-inflammatory drugs,” and “opioids.” The detailed search strategies are shown in Supplementary Method S1.

### Inclusion and Exclusion Criteria

We screened relevant RCT studies according to the following criteria: 1) all the patients with acute renal colic involved in the study were older than 16 years; 2) patients were administered only NSAIDs and opioids in the treatment of renal colic without a request of dose or route of drugs; 3) sufficient data were available about outcomes such as changes in the visual analog scale (VAS) or numerical rating scale (NRS) at 0–30 min, the VAS or NRS over different time periods (at 15, 30, 45, 60 min), need for rescue analgesia, and adverse events involving vomit and dizziness.

Patients' pain was assessed by using the VAS or NRS, which rates the amount of pain from 0 to 10, where 0 means no pain and 10 means the most pain. The adverse drug reactions include the overall incidence of adverse drug events and the incidence of dizziness and vomiting.

The exclusion criteria were as follows: 1) participants presenting to the hospital because of acute renal colic with more than 12 h after disease onset; 2) duplicate studies; 3) data were not available.

### Data Extraction

Titles and abstracts of the articles were screened according to the preset inclusion and exclusion criteria. Subsequently, the eligible studies were registered through reading of the full text of the articles. Two authors (Xie-Yuan Leng and Chang-Ning Liu) individually collected the data and information from included studies involving research design, the average age, the proportion of gender, intervention measures, the comparison of analgesic effect, the route of administration, and adverse events. We contacted the original author for inquiry of the missing data. Additionally, two outcomes that changed in the VAS at 0–30 min and the VAS over different time periods (at 15, 30, 45, and 60 min) were defined as the primary outcomes, and the other outcomes were considered as secondary outcomes in this systematic review and meta-analysis. Besides, the scores of 100 subscales were converted into 10 subscales, so as to achieve the unity of data units. Any discrepancies were resolved in discussions with the third reviewer (Hao-Dong Peng).

### Quality Assessment

Two reviewers (Xie-Yuan Leng and Chang-Ning Liu) independently completed quality assessment of included RCTs by adopting the Cochrane Collaboration's tool, which involves seven items that evaluated quality via ranking “low risk of bias,” “unclear risk of bias,” or “high risk of bias.”

### Statistical Analysis

The measurement tools for pain used in the included studies included the VAS and NRS of 100 mm and 10 cm, with 100–0 and 10–0, respectively, to indicate the most severe pain to no pain. In this study, the scores of 100 subscales were converted into 10 subscales, so as to achieve the unity of data units. Dichotomous outcomes were expressed as relative risks (RR) with 95% confidence interval (CI), and continuous outcomes were expressed as mean difference (MD) with 95% CI (Deeks, 2002; Cumpston et al., 2019). We applied Knapp–Hartung adjustments to reduce the uncertainty of inter-study heterogeneity estimation. The combined data were processed first and the uncertainty heterogeneity within the study was reduced. The subgroup analysis was conducted to compare the NSAIDs to opioids, so as to analyze the source of heterogeneity according to the different routes of administration, NSAID type, and opioids type on patients. The primary outcome was assessed according to the Grading of Recommendations Assessment, Development and Evaluation (GRADE) framework based on five items including the risk of bias, inconsistency, indirectness, imprecision, and publication bias (Guyatt et al., 2008). The funnel plots were used to evaluate the possible publication bias of the efficacy and safety of the drug. The Preferred Reporting Items for Systematic Reviews and Meta-Analyses (PRISMA) statement for the reporting of systematic reviews and meta-analysis was employed (Liberati et al., 2009).

## Results

### Literature Search and Characteristics of Included Studies

**FIGURE 1:**
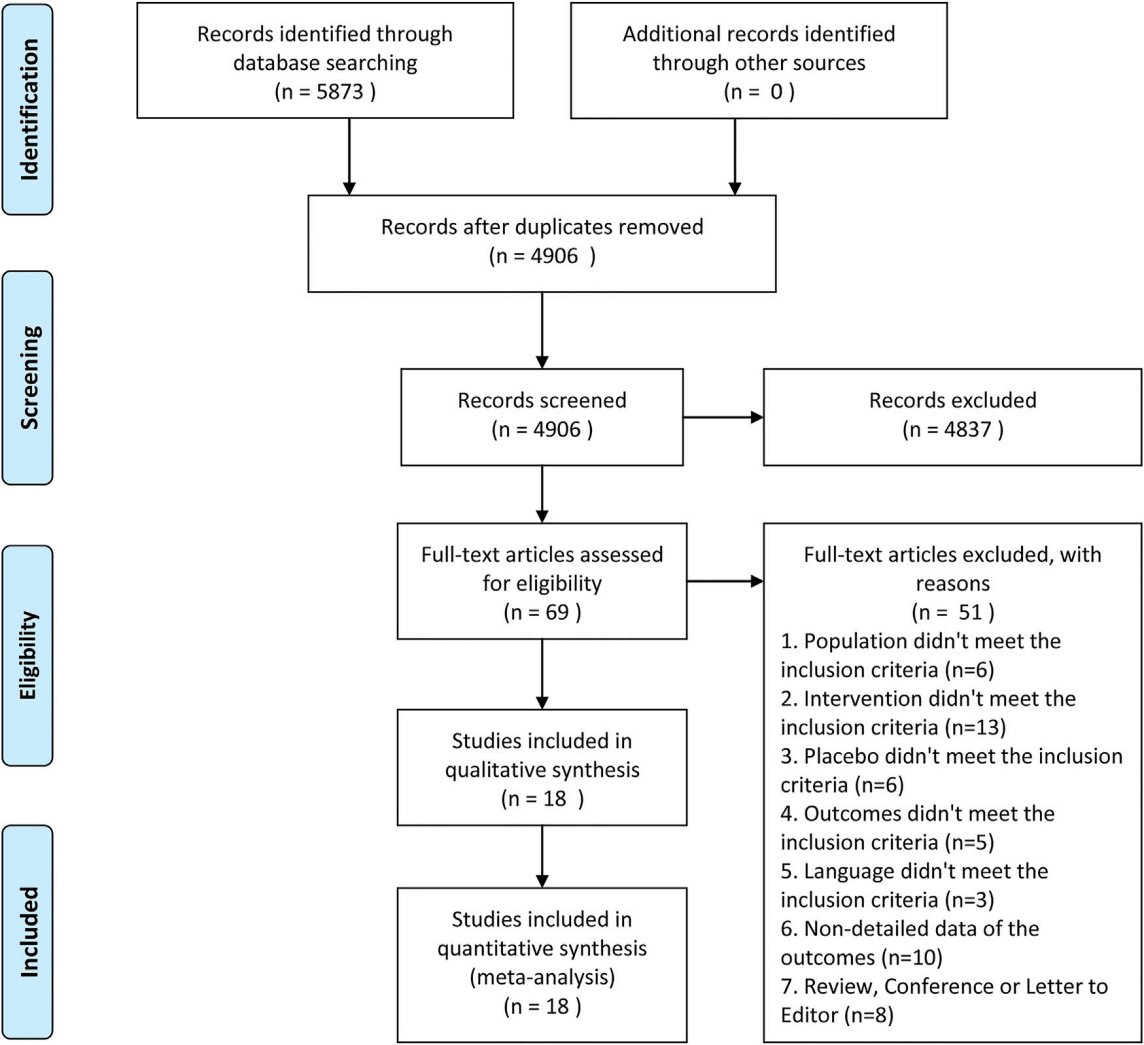
Summary of trial identification and selection.

There were 5,873 articles obtained from the initially screened databases, and finally, a total of 18 studies (Hetherington and Philp, 1986; Thompson et al., 1989; Oosterlinck et al., 1990; Sandhu et al., 1994; Cordell et al., 1996; Larkin et al., 1999; Safdar et al., 2006; Bektas et al., 2009; Serinken et al., 2012; Soleimanpour et al., 2012; Azizkhani et al., 2013; Ay et al., 2014; Masoumi et al., 2014; Pathan et al., 2016a; Zamanian et al., 2016; Al et al., 2018; Sotoodehnia et al., 2019; Rezaei et al., 2020) were identified, including 3,121 participants, who met eligible criteria in this systematic review and meta-analysis through the process of deleting duplicate articles, and removing ineligible studies with reasons. All the details of screening the articles are illustrated in Figure 1. In addition, Table 1 demonstrates basic information of the included studies, mainly consisting of a summary of patient characteristics, administration of drugs (NSAIDs and opioids), and outcomes.

**TABLE 1:** Summary of included clinical trials and patient characteristics.

Study	Year	Population	Sample size	Male (%)	Mean age ± SD	Route of administration	NSAIDs	Opioids	Outcomes	Assessment tool
Al	2018	Turk	300	DKT 78 (78%)/fentanyl 71 (71%)/paracetamol 67 (67%)	42.2	NR	IV dexketoprofen/trometamol and paracetamol	IV fentanyl	1) Pain score (VAS 10 cm) at 30 min 2) Need for rescue analgesia 3) Adverse events	VAS
Ay	2014	Turk	52	NR	NR	Injection	IV dexketoprofen/trometamol	IV meperidine hydrochloride	1) Pain score (NRS-11) at 30 min 2) Need for rescue analgesia 3) Adverse events	NRS
Azizkhani	2013	Iranian	124	84 (67.7%)	38.40 ± 11.60/39.73 ± 11.62	IV injection	IV acetaminophen	IV morphine	1) Pain score (VAS 10 cm) at 30 min 2) Adverse events	VAS
Bektas	2009	Turk	95	31 (67%)/27 (55%)	35 ± 10/39 ± 11	IV injection	Paracetamol 1 g in 100 ml normal saline solution	Morphine 0.1 mg/kg in 100 ml normal saline solution	1) Pain score (VAS 10 cm) at 30 min 2) Need for rescue analgesia 3) Adverse events	VAS
Cordell	1996	American	71	30 (83%)/28 (80%)	38,8_+1.7/42.0 + 1.9	IV injection	Ketorolac 60 mg	Meperidine 50 mg	1) Pain score (VAS 10 cm) at 30 min 2) Need for rescue analgesia 3) Adverse events	VAS
Hetherington	1986	British	58	NR	NR	IM injection	Diclofenac 75 mg	Pethidine 100 mg	1) Need for rescue analgesia	VAS
Larkin	1999	American	70	26 (79%)/27 (73%)	45.5 ± 16/40.7 ± 13.3	NR	IM dose of 60 mg of ketorolac	A single weight dependent dose of IM meperidine (patients weighing from 50 to 90 kg received 100 mg of meperidine while those weighing more than 90 kg received 150 mg of meperidine	1) Need for rescue analgesia 2) Adverse events	VAS
Masoumi	2014	Iranian	108	43 (79.6%)/39 (72.2%)	36.07 ± 9.7/34.96 ± 8.94	IV injection	Acetaminophen (IV acetaminophen with a dose of 1 g in 100 ml normal saline)	Morphine (0.1 mg/kg morphine in 100 ml normal saline was infused)	1) Pain score (NRS-11) at 30 min2) Adverse events	VAS
Oosterlinck	1990	American	111	NR	NR	IM injection	Single IM doses of 10 mg (1 ml of 1% solution) or 90 mg (3 ml of 3% solution) of ketorolac	100 mg (2 ml of 5% solution) of pethidine	1) Pain score (VAS 10 cm) at 30 min 2) Adverse events	VAS
Pathen	2016	Qatari	1095	NR	NR	Diclofenac IM/acetaminophen IV/morphine IV	Diclofenac 75 mg/acetaminophen 1 g	Morphine 0.1 mg/kg	1) Need for rescue analgesia2) Adverse events	VAS
Rezaei	2020	Iranian	186	70 (75.3%)/69 (70.4%)	45.56 ± 12.33/42.33 ± 13.92	IM injection/Atomization inhalation	IV ketorolac	Nebulized fentanyl	1) Pain score (VAS 10 cm) at 30 min	NRS
Safdar	2006	American	86	29 (67%)/29 (67%)	39.3 ± 9.9/37.3 ± 10	IV injection	Ketorolac 15 mg	Morphine 5 mg	1) Pain score (VAS 10 cm) at 30 min 2) Need for rescue analgesia 3) Adverse events	VAS
Sandhu	1994	Britisher	110	15 (46.9%)/58 (74.4%)	45.2 ± 14.6/42.1 ± 14.6	IM injection	Single 30 mg IM dose of ketorolac	IM pethidine 100 mg	1) Pain score (VAS 10 cm) at 30 min 2) Need for rescue analgesia 3) Adverse events	VAS
Serinken	2012	NR	73	51 (70%)	30.2 ± 8.6	IV injection	IV single-dose paracetamol	IV single-dose morphine	1) Pain score (VAS 10 cm) at 30 min 2) Need for rescue analgesia 3) Adverse events	VAS
Sotoodehnia	2019	Iranian	126	52 (81.2%)/44 (71%)	37.9 ± 10.6/34.2 ± 9.9	IM injection	IV ketorolac	IV ketamine	1) Pain score (VAS 10 cm) at 30 min 2) Adverse events	NRS
Thompson	1989	Britisher	58	NR	NR	Rectal administration/injection	Rectal diclofenac	Pethidine injection	1) Complete pain relief at 30 min 2) Adverse events	VAS
Zamanian	2016	Iranian	158	52/50	37.3 ± 11.5/37.2 ± 10.6	NR	100 mg indomethacin suppository	10 mg morphine suppository	1) Pain score (NRS-11) at 40 min 2) Adverse events	VAS
Soleimanpour	2012	Iranian	240	86 (71.7%)/90 (75%)	35.23 ± 12.37/37.71 ± 11.08	Single-dose IV	Single-dose IV lidocaine (1.5 mg/kg)	Single-dose IV morphine (0.1 mg/kg)	1) Pain score (VAS 10 cm) at 30 min 2) Adverse events	VAS

NR, not reported.

### Quality Assessment of Included Studies

A total of 18 included studies in this systematic review and meta-analysis were rated according to the Cochrane Collaboration's tool in Supplementary Figure S1.

### Primary Outcomes

#### Changes in Visual Analog Scale at 0–30 min

**FIGURE 2:**
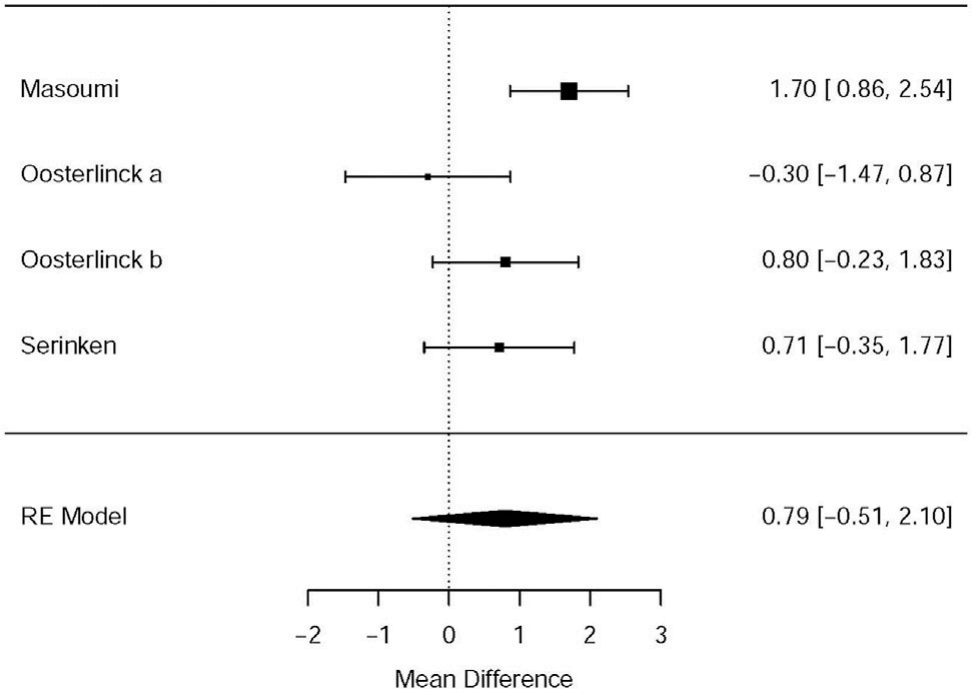
The results of meta-analysis for changes in pain scores (VAS) at 0–30 min.

Three RCTs (Oosterlinck et al., 1990; Serinken et al., 2012; Masoumi et al., 2014) presented the outcome that the changes of the VAS at the 30th minute after applying NSAIDs and opioids as the treatment of acute renal colic. Based on these included studies, no marked significance between the NSAIDs group and opioids group was observed in terms of the analgesic effect for patients with renal colic (MD = 0.79, 95% CI: −0.51, 2.1, *I* ^2^ = 61%, NSAIDs vs. opioids). More data about the changes in the VAS at the 30th min are presented in Figure 2. The overall evidence of changes of the VAS at the 30th min was assessed to be of low quality using the GRADE assessment framework (Table 2).

**TABLE 2:** GRADE profile of changes in VAS at 0–30 min.

Quality assessment						Summary of findings			Quality of evidence
Outcomes	Risk of bias	Inconsistency	Indirectness	Imprecision	Publication bias	Sample			
						With NSAIDs	With opioids	MD, 95% CI	
Changes in VAS at 0–30 min	No serious limitations	Serious limitations due to the inconsistency^a^	No serious limitations	Serious limitations due to the imprecision^b^	No serious limitations	166	163	0.79 (−0.02, 1.61)	⊕⊕○○ Low, Due to the inconsistency and imprecision

a The test for heterogeneity is significant, and the *I* ^2^ is moderate, 44%.

b The 95% CI for the absolute effects include clinical benefit and no benefit, and the sample size was insufficient.

**FIGURE 3:**
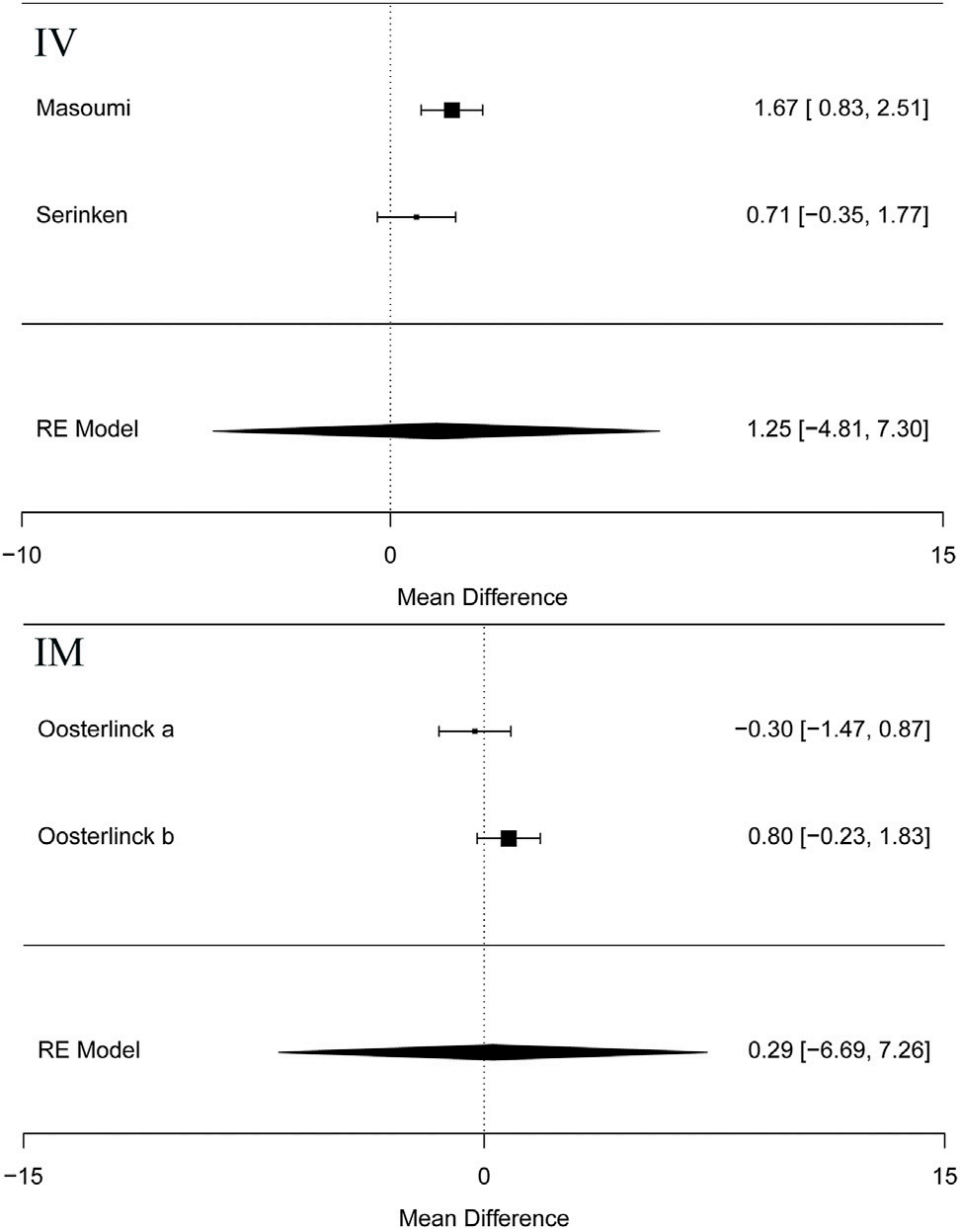
The results of meta-analysis for changes in pain scores (VAS) at 0–30 min based on different routes.

Besides, the information of the subgroup analysis by the different routes of administration and NSAID type are presented in Figure 3. The results presented (Serinken et al., 2012; Masoumi et al., 2014) that opioids in the form of IV administration didn't have significant difference on changes in the VAS at 0–30 min in the renal colic patients, when compared with the NSAIDs through the same route (MD = 1.25, 95% Cl: −4.81, 7.30, *I* ^2^ = 48%, NSAIDs vs. opioids) (Figure 3). Conversely, no statistical significance was evident with the primary outcome (Oosterlinck et al., 1990) in comparison between the NSAID and opioid groups when administered IM (MD = 0.29, 95% Cl: −6.69, 7.26, *I* ^2^ = 48%, NSAIDs vs. opioids) (Figure 3).

Based on the subgroup analysis of different NSAIDs and opioids in Table 3, it is shown that the changes in the VAS at 0–30 min had no statistical difference (MD = 0.29, 95% CI: −6.69, 7.26, *I* ^2^ = 48%, NSAIDs vs. opioids) between ketorolac and pethidine, provided by the data of two RCTs of Oosterlinck et al. (1990). However, when compared with morphine from only one study (Masoumi et al., 2014), acetaminophen (MD = 1.70, 95% CI: 0.86, 2.54, *I* ^2^ = NA, NSAIDs vs. opioids) can significantly lower the changes in the VAS at 0–30 min, but paracetamol (MD = 0.71, 95% Cl: −0.35, 1.77, *I* ^2^ = NA, NSAIDs vs. opioids) demonstrated no significant difference (Table 3).

**TABLE 3:** Subgroup analysis of NSAIDs and opioids of the changing in VAS at 0–30 min.

Study	Year	NSAIDs mean	NSAIDs SD	NSAIDs total	Opioids mean	Opioids SD	Opioids total	Medicine NSAIDs	Medicine opioids	MD (95%CI) NSAIDs vs. opioids	*I* ^2^ (%)
Masoumi	2014	4.65	2.25	54	2.95	2.18	54	Acetaminophen	Morphine	1.7 (0.86, 2.54)	NA
Oosterlinck a	1990	5.4	2.6	39	5.7	2.6	37	Ketorolac	Pethidine	0.29 (−6.69, 7.26)	47.83
Oosterlinck b	1990	6.5	1.8	35	5.7	2.6	37	Ketorolac	Pethidine	0.29 (−6.69, 7.26)	47.83
Serinken	2012	6.37	2.17	38	5.66	2.44	35	Paracetamol	Morphine	0.71 (−0.35, 1.77)	NA

#### VAS Over Different Time Periods

**FIGURE 4:**
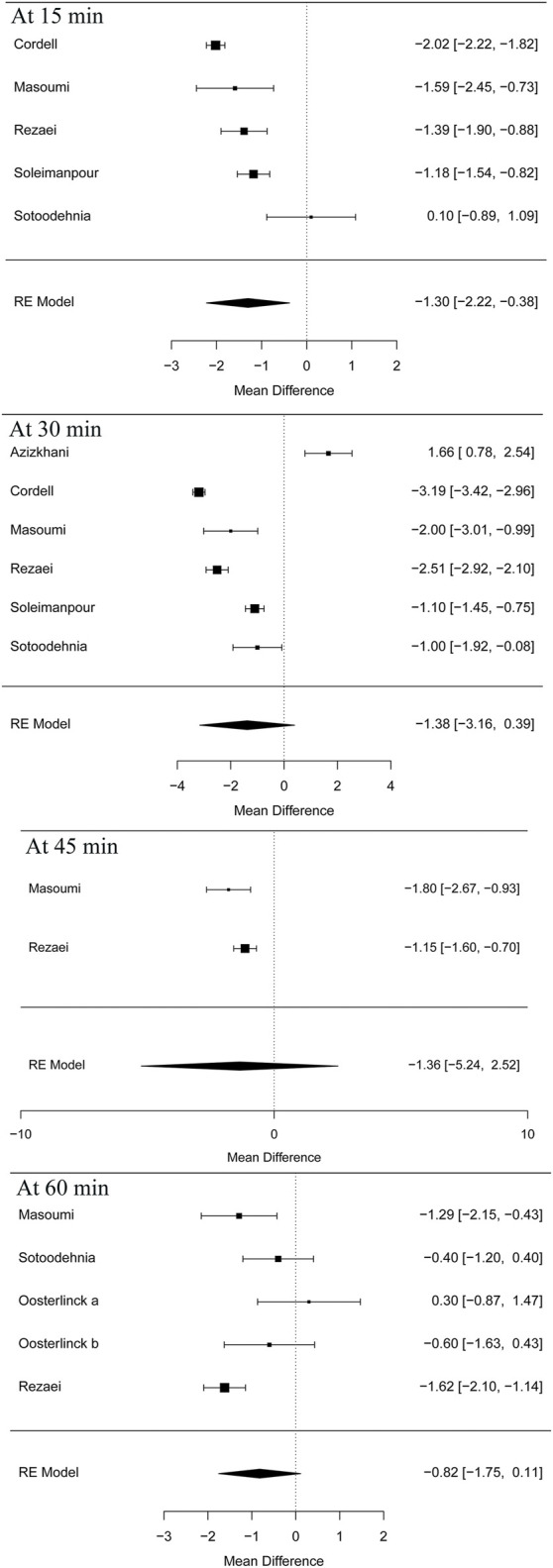
The results of meta-analysis for pain scores (VAS) over different time periods.

**FIGURE 5:**
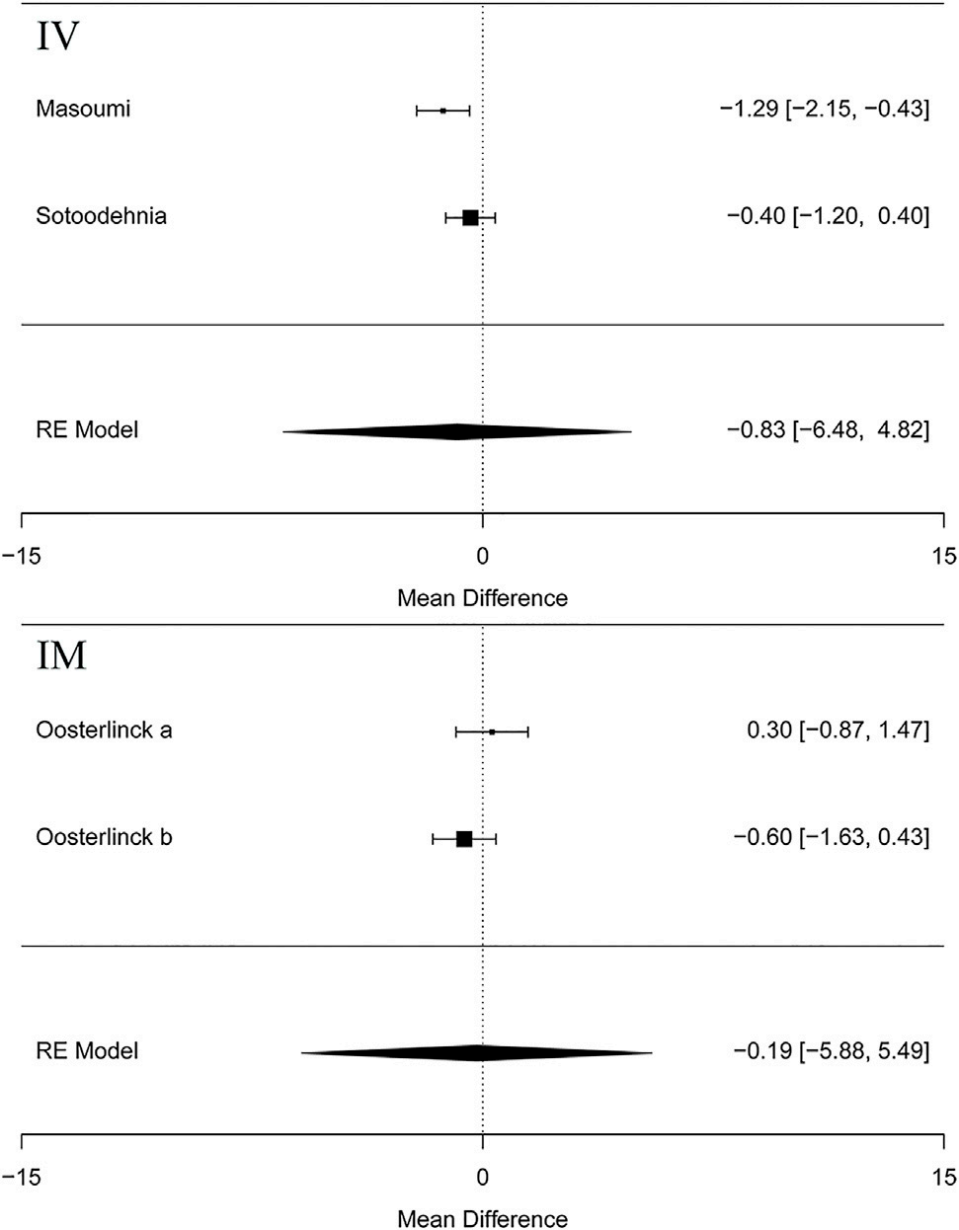
The results of meta-analysis for pain scores (VAS) at 60 min based on different routes.

Five articles (Cordell et al., 1996; Masoumi et al., 2014; Sotoodehnia et al., 2019; Rezaei et al., 2020) in this meta-analysis presented the result of the VAS at 15 min (MD = −1.30 95% CI: −2.22, −0.38, *I* ^2^ = 88.8%, NSAIDs vs. opioids) and demonstrated that the NSAIDs group greatly improved former outcomes compared to the opioids group, as is shown in Figure 4. However, the VAS at 30 min (Cordell et al., 1996; Soleimanpour et al., 2012; Azizkhani et al., 2013; Masoumi et al., 2014; Sotoodehnia et al., 2019; Rezaei et al., 2020) (MD = −1.38, 95% CI: −3.16, 0.39, *I* ^2^ = 98%, NSAIDs vs. opioids) indicated that there was no significant difference between the opioid and NSAID groups. Moreover, similar results were also seen in the VAS at 45 min (Masoumi et al., 2014; Rezaei et al., 2020) (MD = −1.36, 95% CI: −5.24, 2.52, *I* ^2^ = 40.7%, NSAIDs vs. opioids) (Figure 4). Besides, the outcome of the VAS at 60 min involved data (Oosterlinck et al., 1990; Masoumi et al., 2014; Sotoodehnia et al., 2019; Rezaei et al., 2020) from both forms of IV and IM administration, and the total result of this outcome shows that using NSAIDs had a similar benefit to using opioids in the treatment of renal colic (MD = −0.82, 95% CI: −1.75, 0.11, *I* ^2^ = 72%, NSAIDs vs. opioids) (Figure 4). Figure 5 shows the details of the VAS over different time periods. Regarding the subgroup analysis of the VAS at 60 min (Figure 5), there was no significant difference between the NSAID and opioid groups in the treatment of renal colic (MD = −0.83, 95% CI: −6.48, 4.82, *I* ^2^ = 47.8%, NSAIDs vs. opioids) (Masoumi et al., 2014; Sotoodehnia et al., 2019). This is similar to the primary outcome in the form of IM (Oosterlinck et al., 1990) (MD = −0.19, 95% CI: −5.88, 5.49, *I* ^2^ = 48.4%, NSAIDs vs. opioids) (Figure 5).

At 15 min, according to the subgroup analysis of different NSAIDs and opioids shown in Table 4, it is not difficult to find that using ketorolac is better than meperidine as per one study (Cordell et al., 1996) (MD = −2.02, 95% CI: −2.22, −1.82, *I* ^2^ = NA, NSAIDs vs. opioids). Three other studies (Soleimanpour et al., 2012; Masoumi et al., 2014; Rezaei et al., 2020) also proved that acetaminophen performed better than morphine (MD = −1.59, 95% CI: −2.45, −0.73, *I* ^2^ = NA, NSAIDs vs. opioids), ketorolac better than fentanyl (MD = −1.39, 95% CI: −1.90, −0.88, *I* ^2^ = NA, NSAIDs vs. opioids), and lidocaine better than morphine (MD = −1.18, 95% CI: −1.54, −0.82, *I* ^2^ = NA, NSAIDs vs. opioids). However, one study showed that there was no significant difference between ketorolac and ketamine at this time (Sotoodehnia et al., 2019).

**TABLE 4:** Subgroup analysis of NSAIDs and opioids of the VAS over different time periods.

Study	Year	NSAIDs mean	NSAIDs SD	NSAIDs total	Opioids mean	Opioids SD	Opioids total	Medicine NSAIDs	Medicine opioids	MD (95%CI) NSAIDs vs. opioids	*I* ^2^ (%)
Cordell	1996	3.48	0.45	36	5.5	0.43	35	Ketorolac	Meperidine	−2.02 (−2.22, −1.82)	NA
Masoumi	2014	5.87	2	54	7.46	2.51	54	Acetaminophen	Morphine	−1.59 (−2.45, −0.73)	NA
Rezaei	2020	5.7	1.75	93	7.09	1.8	93	Ketorolac	Fentanyl	−1.39 (−1.9, −0.88)	NA
Soleimanpour	2012	1.37	1.32	120	2.55	1.52	120	Lidocaine	Morphine	−1.18 (−1.54, −0.82)	NA
Sotoodehnia	2019	4.8	2.5	64	4.7	3.1	62	Ketorolac	Ketamine	0.1 (−0.89, 1.09)	NA
At 15 min											
Azizkhani	2013	2.41	3.29	62	0.75	1.31	62	Acetaminophen	Morphine	−0.16 (−23.41, 23.09)	NA
Cordell	1996	2.47	0.46	36	5.66	0.52	35	Ketorolac	Meperidine	−3.19 (−3.42, −2.96)	NA
Masoumi	2014	4.09	2.68	54	6.09	2.69	54	Acetaminophen	Morphine	−0.16 (−23.41, 23.09)	NA
Rezaei	2020	3.95	1.32	93	6.46	1.55	93	Ketorolac	Fentanyl	−2.51 (−2.92, −2.1)	NA
Soleimanpour	2012	1.13	1.15	120	2.23	1.57	120	Lidocaine	Morphine	−1.1 (−1.45, −0.75)	NA
Sotoodehnia	2019	1.8	2.3	64	2.8	2.9	62	Ketorolac	Ketamine	−1 (−1.92, −0.08)	NA
At 30 min											
Masoumi	2014	2.46	2.09	54	4.26	2.51	54	Acetaminophen	Morphine	−1.8 (−2.67, −0.93)	NA
Rezaei	2020	3.73	1.37	93	4.88	1.75	93	Ketorolac	Fentanyl	−1.15 (−1.6, −0.7)	NA
At 45 min											
Masoumi	2014	2.02	2.03	54	3.31	2.51	54	Acetaminophen	Morphine	−1.29 (−2.15, −0.43)	NA
Oosterlinck a	1990	2.6	2.6	39	2.3	2.6	37	Ketorolac	Pethidine	−0.19 (−5.88, 5.49)	NA
Oosterlinck b	1990	1.7	1.8	35	2.3	2.6	37	Ketorolac	Pethidine	−0.19 (−5.88, 5.49)	NA
Rezaei	2020	3.18	1.35	93	4.8	1.91	93	Ketorolac	Fentanyl	−1.62 (−2.1, −1.14)	NA
Sotoodehnia	2019	1	2.2	64	1.4	2.4	62	Ketorolac	Ketamine	−0.4 (−1.2, −0.4)	NA
At 60 min											

Similarly, four articles (Cordell et al., 1996; Soleimanpour et al., 2012; Sotoodehnia et al., 2019; Rezaei et al., 2020) as shown in Table 4 reported the indicated significant differences on the VAS at 30 min for subjects with acute renal colic. They used ketorolac–meperidine (MD = −3.19, 95% CI: −3.42, −2.96, *I* ^2^ = NA, NSAIDs vs. opioids), ketorolac–fentanyl (MD = −2.51, 95% CI: −2.92, −2.1, *I* ^2^ = NA, NSAIDs vs. opioids), lidocaine–morphine (MD = −1.1, 95% CI: −1.45, −0.75, *I* ^2^ = NA), and ketorolac–ketamine (MD = −1, 95% CI: −1.92, −0.08, *I* ^2^ = NA, NSAIDs vs. opioids), respectively, and showed that NSAIDs had more advantages in the treatment of acute renal colic at 30 min. However, two RCTs (Azizkhani et al., 2013; Masoumi et al., 2014) showed that there was no significant difference between acetaminophen and morphine at this time (MD = −0.16, 95% CI: −23.41, 23.09, *I* ^2^ = NA, NSAIDs vs. opioids).

It happens that there is a similar case, at 45 min; two studies (Masoumi et al., 2014; Rezaei et al., 2020) showed the superiority of acetaminophen over morphine (MD = −1.8, 95% CI: −2.67, −0.93, *I* ^2^ = NA, NSAIDs vs. opioids) and ketorolac over fentanyl (MD = −1.15, 95% CI: −1.6, −0.7, *I* ^2^ = NA, NSAIDs vs. opioids).

Additionally, with regard to the subgroup analysis by NSAIDs and opioids, at 60 min, a study (Oosterlinck et al., 1990) showed that there was no significant difference between ketorolac and pethidine (MD = −0.19, 95% CI: −5.88, 5.49, *I* ^2^ = NA, NSAIDs vs. opioids). However, three other studies (Masoumi et al., 2014; Sotoodehnia et al., 2019; Rezaei et al., 2020) that used acetaminophen–morphine (MD = −1.29, 95% CI: −2.15, −0.43, *I* ^2^ = NA, NSAIDs vs. opioids), ketorolac–fentanyl (MD = −1.62, 95% CI: −2.10, −1.14, *I* ^2^ = NA, NSAIDs vs. opioids), and ketorolac–ketamine (MD = −0.40, 95% CI: −1.20, −0.40, *I* ^2^ = NA, NSAIDs vs. opioids), respectively, showed that NSAIDs were more effective than opioids (Table 4 **)**.

### Secondary Outcomes

#### Need for Rescue Analgesia

**FIGURE 6:**
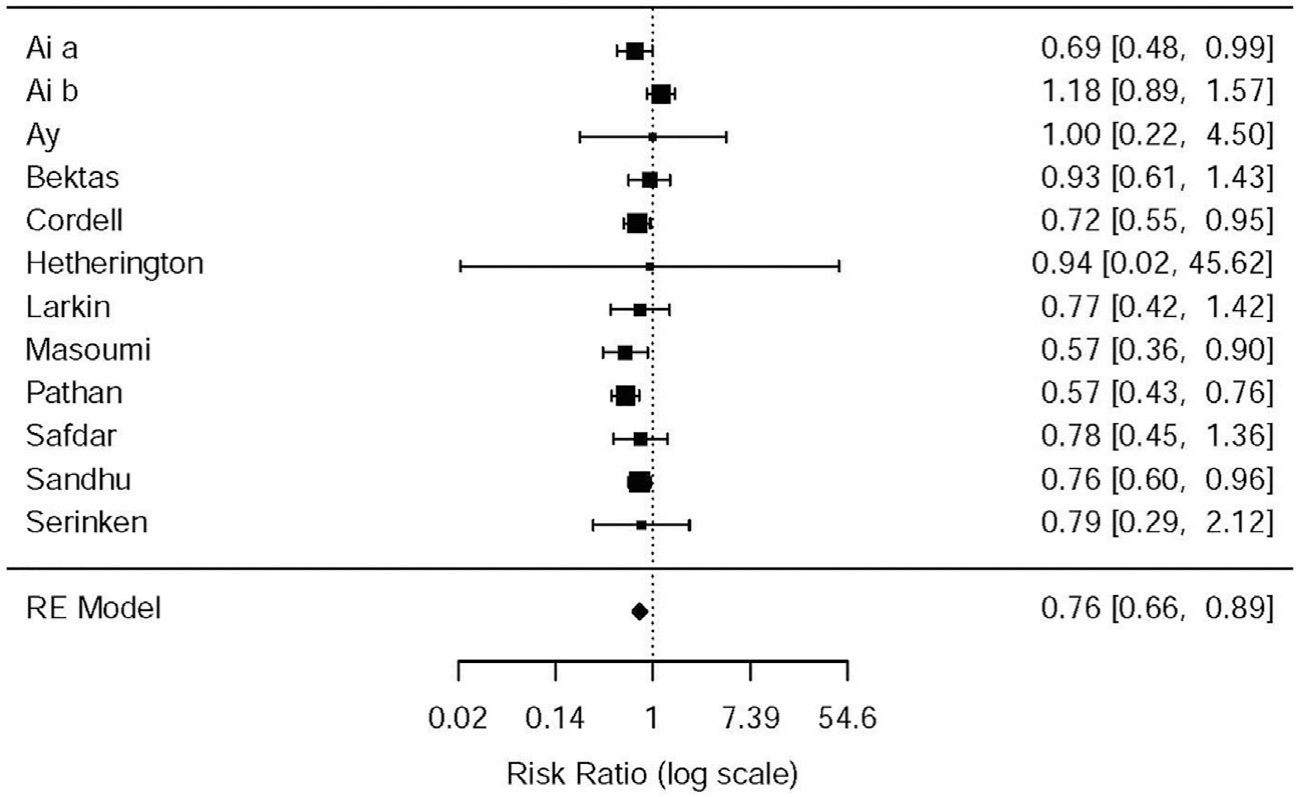
The results of meta-analysis for need for rescue analgesia.

In the light of the 11 included studies (Hetherington and Philp, 1986; Sandhu et al., 1994; Cordell et al., 1996; Larkin et al., 1999; Safdar et al., 2006; Bektas et al., 2009; Serinken et al., 2012; Ay et al., 2014; Masoumi et al., 2014; Pathan et al., 2016a; Al et al., 2018), there were a total of 2,262 patients who needed rescue analgesia during the therapy of acute renal colic. The difference among nine RCTs, to be exact, involved the standards of rescue, administration of NSAIDs or opioids (e.g., dose of drugs, time of using analgesic) and the criteria that rescue succeed. The difference was observed with data from the above studies that opioids were more likely to receive later rescue when they were used for analgesia (RR = 0.76, 95% CI: 0.66, 0.89, *I* ^2^ = 42.3%, NSAIDs vs. opioids). The relevant data about the need for rescue analgesia is presented in Figure 6 (please refer for details).

Some studies were performed by the subgroup analysis on the NSAID and Opioid types. Three studies (Sandhu et al., 1994; Masoumi et al., 2014; Pathan et al., 2016b) used morphine–acetaminophen (RR = 0.57, 95% CI: 0.36, 0.9, *I* ^2^ = NA, NSAIDs vs. opioids); morphine–diclofenac (RR = 0.57, 95% CI: 0.43,0.76, *I* ^2^ = NA, NSAIDs vs. opioids), and pethidine–ketorolac (RR = 0.76, 95% CI: 0.6, 0.96, *I* ^2^ = NA, NSAIDs vs. opioids), respectively; all of them showed that morphine is easier to use for rescue analgesia than NSAIDs. Several other studies, as given in Table 5, showed no significant statistical difference between the NSAIDs and opioids.

**TABLE 5:** Subgroup analysis of NSAIDs and opioids of the needing for rescue analgesia.

Study	Year	NSAIDs events	NSAIDs total	Opioids events	Opioids total	Medicine NSAIDs	Medicine opioids	RR (95%CI) NSAIDs vs. opioids	*I* ^2^ (%)
Ai a	2018	31	100	45	100	Dexketoprofen	Fentanyl	0.69 (0.48, 0.99)	NA
Ai b	2018	53	100	45	100	Paracetamol	Fentanyl	1.18 (0.89, 1.57)	NA
Ay	2014	3	26	3	26	Dexketoprofen	Meperidine	1 (0.22, 4.5)	NA
Bektas	2009	21	46	24	49	Paracetamol	Morphine	0.91 (0.42, 1.95)	NA
Cordell	1996	23	36	31	35	Ketorolac	Meperidine	0.73 (0.53, 1)	NA
Hetherington	1986	0	30	0	28	diclofenac	Pethidine	0.94 (0.02, 45.62)	NA
Larkin	1999	11	33	16	37	Ketorolac	Meperidine	0.73 (0.53, 1)	NA
Masoumi	2014	17	54	30	54	Acetaminophen	Morphine	0.57 (0.36, 0.9)	NA
Pathan	2016	63	547	111	548	Diclofenac/acetaminophen	Morphine	0.57 (0.43, 0.76)	NA
Safdar	2006	14	43	18	43	Ketorolac	Morphine	0.78 (0.45, 1.36)	NA
Sandhu	1994	43	76	58	78	Ketorolac	Pethidine	0.76 (0.6, 0.96)	NA
Serinken	2012	6	38	7	35	Paracetamol	Morphine	0.91 (0.42, 1.95)	NA

#### Drug-Related Adverse Events

**FIGURE 7:**
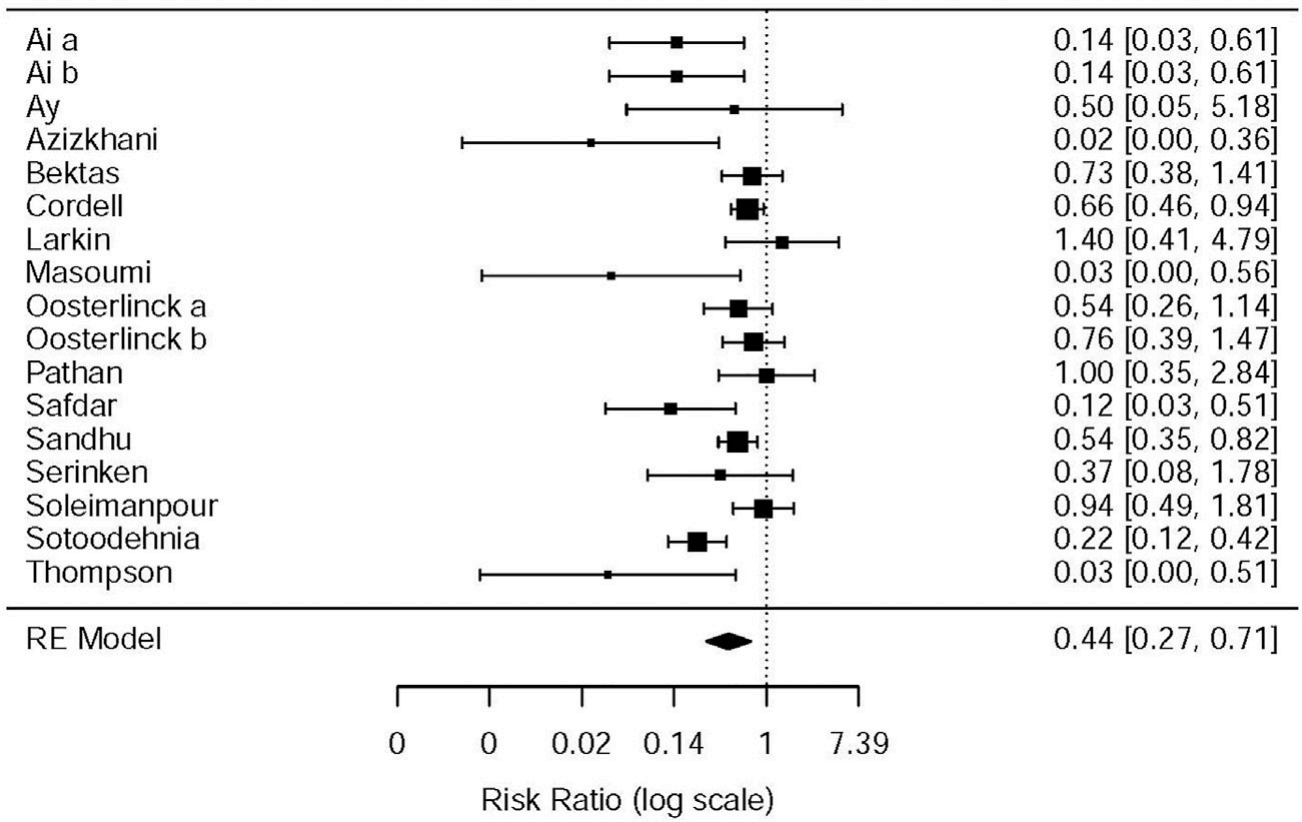
The results of meta-analysis for side-effects caused by the medication.

**FIGURE 8:**
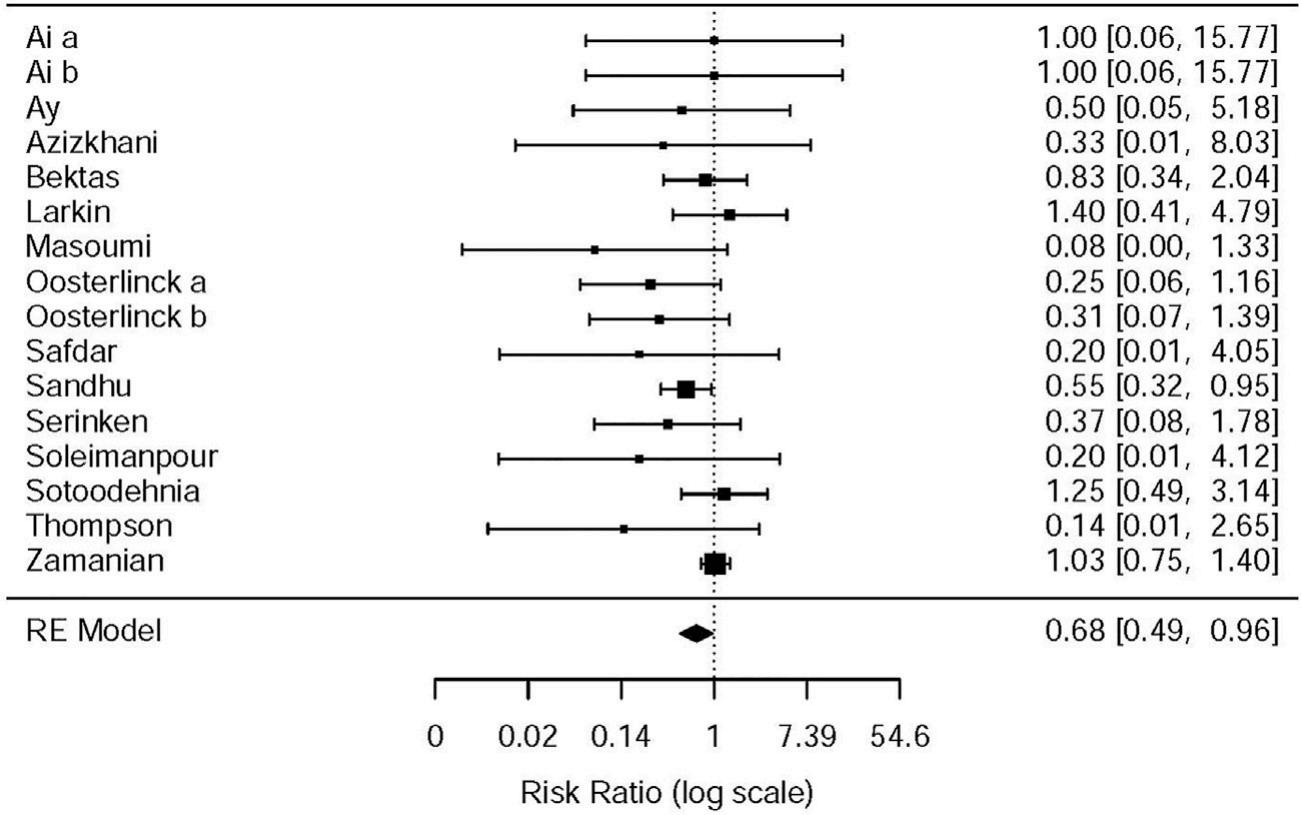
The results of meta-analysis for vomiting caused by the medications.

**FIGURE 9:**
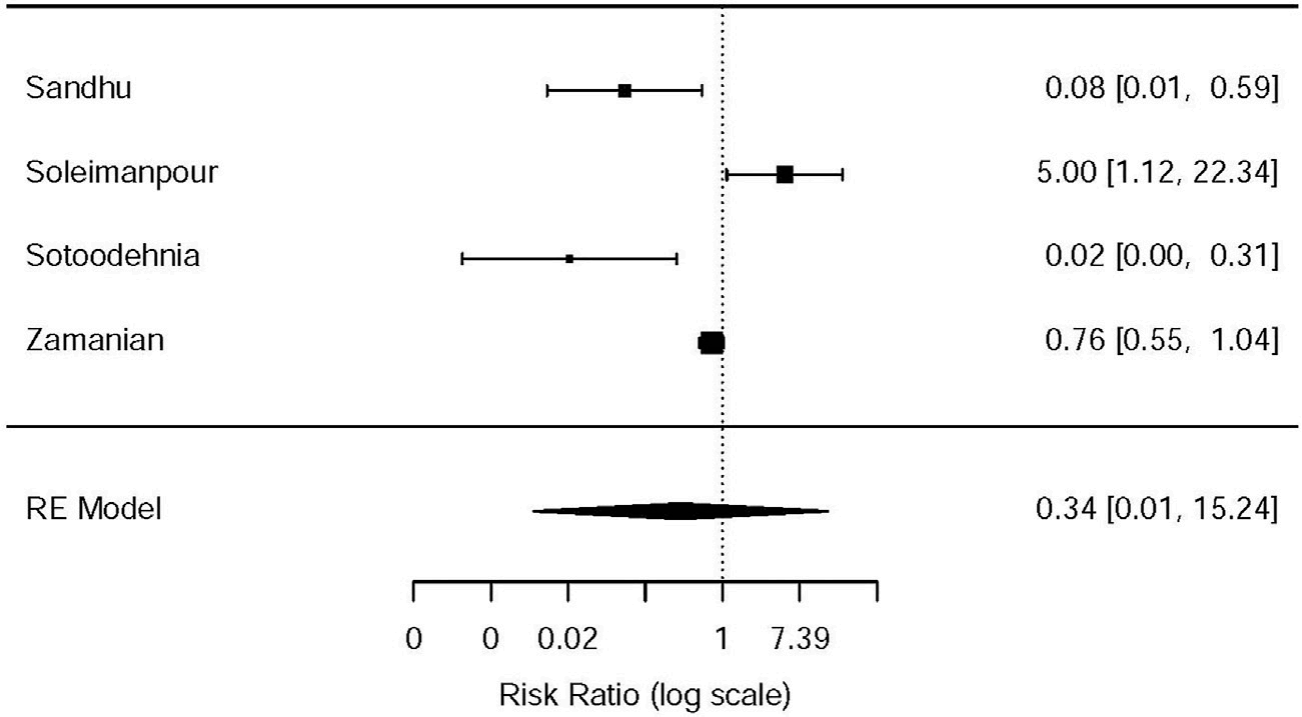
The results of meta-analysis for dizziness caused by the medications.

This systematic review and meta-analysis collected all adverse events described in 15 original articles (Thompson et al., 1989; Oosterlinck et al., 1990; Sandhu et al., 1994; Cordell et al., 1996; Larkin et al., 1999; Safdar et al., 2006; Bektas et al., 2009; Serinken et al., 2012; Soleimanpour et al., 2012; Azizkhani et al., 2013; Masoumi et al., 2014; Ay et al., 2014; Pathan et al., 2016a; Al et al., 2018; Sotoodehnia et al., 2019), which differ in types and standards of adverse events caused by drugs. We synthesized all data about drug-related adverse events and found that it was more possible for opioids to lead to adverse events in the treatment of patients with acute renal colic than the NSAIDs group (RR = 0.44, 95% CI: 0.27, 0.71, *I* ^2^ = 65.7%, NSAIDs vs. opioids) (Figure 7). According to drug-related adverse events, the subgroup analysis was conducted to investigate the difference of both therapeutic regimens in renal colic patients. A total of 15 studies (Thompson et al., 1989; Oosterlinck et al., 1990; Sandhu et al., 1994; Larkin et al., 1999; Safdar et al., 2006; Bektas et al., 2009; Soleimanpour et al., 2012; Serinken et al., 2012; Azizkhani et al., 2013; Masoumi et al., 2014; Ay et al., 2014; Zamanian et al., 2016; Al et al., 2018; Sotoodehnia et al., 2019) referred to vomiting caused by the medications, and there were remarkable differences among the patients of the NSAIDs group (RR = 0.68, 95% CI: 0.49, 0.96, *I* ^2^ = 22.6%, NSAIDs vs. opioids) (Figure 8). However, the participants in the opioids group and NSAIDs group showed no significance in dizziness comparing with those in the NSAIDs group (RR = 0.34, 95% CI: 0.01, 15.24, *I* ^2^ = 90.3%, NSAIDs vs. opioids) on the basis of four RCTs (Sandhu et al., 1994; Soleimanpour et al., 2012; Zamanian et al., 2016; Sotoodehnia et al., 2019) (Figure 9).

The NSAIDs and opioids subgroup analysis was performed on drug-related adverse events as given in Table 6. Two studies (Oosterlinck et al., 1990; Sandhu et al., 1994) showed that pethidine is more likely to produce drug-related adverse events than ketorolac (RR = 0.58, 95% CI: 0.38, 0.90, *I* ^2^ = NA, NSAIDs vs. opioids). The other two studies (Azizkhani et al., 2013; Masoumi et al., 2014) showed the same results between morphine and acetaminophen (RR = 0.03, 95% CI: 0, 0.45, *I* ^2^ = NA, NSAIDs vs. opioids). One study (Thompson et al., 1989) showed that pethidine is more likely to produce drug-related adverse events than diclofenac (RR = 0.03, 95% CI: 0, 0.51, *I* ^2^ = NA, NSAIDs vs. opioids). Furthermore, the study by Safdar et al. (2006) showed that morphine is more likely to produce drug-related adverse events than ketorolac (RR = 0.12, 95% CI: 0.03, 0.51, *I* ^2^ = NA, NSAIDs vs. opioids). The study by Sotoodehnia et al. (2019) showed that ketamine is more likely to produce drug-related adverse events than ketorolac (RR = 0.22, 95% CI: 0.12, 0.42, *I* ^2^ = NA, NSAIDs vs. opioids). However, there are still seven studies (Cordell et al., 1996; Larkin et al., 1999; Bektas et al., 2009; Serinken et al., 2012; Soleimanpour et al., 2012; Ay et al., 2014; Pathan et al., 2016b) that indicated that there was no significant statistical difference in drug side effects between the opioids and NSAIDs (Table 6). All in all, most of the included studies showed that opioids were more prone to drug side effects.

**TABLE 6:** Subgroup analysis of NSAIDs and opioids of the drug-related adverse events.

Study	Year	NSAIDs events	NSAIDs total	Opioids events	Opioids total	Medicine NSAIDs	Medicine opioids	RR (95%CI) NSAIDs vs. opioids	*I* ^2^ (%)
Ai a	2018	2	100	14	100	Dexketoprofen	Fentanyl	0.14 (0.03, 0.61)	NA
Ai b	2018	2	100	14	100	Paracetamol	Fentanyl	0.14 (0.03, 0.61)	NA
Sotoodehnia	2019	9	64	39	62	Ketorolac	Ketamine	0.22 (0.12, 0.42)	NA
Ay	2014	1	26	2	26	Dexketoprofen	Meperidine	0.5 (0.05, 5.18)	NA
Cordell	1996	19	36	28	35	Ketorolac	Meperidine	0.76 (0.02, 30.52)	NA
Larkin	1999	5	33	4	37	Ketorolac	Meperidine	0.76 (0.02, 30.52)	NA
Azizkhani	2013	0	62	22	62	Acetaminophen	Morphine	0.03 (0, 0.45)	NA
Masoumi	2014	0	54	14	54	Acetaminophen	Morphine	0.03 (0, 0.45)	NA
Pathan	2016	7	547	7	548	Diclofenac/acetaminophen	Morphine	1 (0.35, 2.84)	NA
Safdar	2006	2	43	16	43	Ketorolac	Morphine	0.12 (0.03, 0.51)	NA
Soleimanpour	2012	15	120	16	120	Lidocaine	Morphine	0.94 (0.49, 1.81)	NA
Bektas	2009	11	46	16	49	Paracetamol	Morphine	0.66 (0.03, 14.58)	NA
Serinken	2012	2	38	5	35	Paracetamol	Morphine	0.66 (0.03, 14.58)	NA
Thompson	1989	0	29	15	29	Diclofenac	Pethidine	0.03 (0, 0.51)	NA
Oosterlinck a	1990	8	39	14	37	Ketorolac	Pethidine	0.58 (0.38, 0.9)	NA
Oosterlinck b	1990	10	35	14	37	Ketorolac	Pethidine	0.58 (0.38, 0.9)	NA
Sandhu	1994	21	76	40	78	Ketorolac	Pethidine	0.58 (0.38, 0.9)	NA

#### Publication Bias

According to the assessment of funnel plots, no obvious publication biases were found as shown in Supplementary Figures S2–S9.

## Discussion

According to a recent study, a large proportion of people suffer from acute renal colic each year, suffering unbearable pain that is described as agony of obstruction in the upper urinary tract by kidney stone(s) (Wang et al., 2020). Unfortunately, there are a myriad of patients around the world who do not adequately receive pain relief, with possible reasons of excessive regulatory restrictions (Minozzi et al., 2013). With respect to therapy of acute renal colic, NSAIDs are prescribed for patients with acute pain and have been identified as necessary agents for treating pain when clinicians made the diagnosis of renal colic, in addition to opioids (Bultitude and Rees, 2012). However, the therapeutic regimen of renal colic is still controversial, with analgesic drug as the appropriate choice for patients who need it to release their pain immediately (Zamanian et al., 2016; Zhili et al., 2020). Therefore, the aim of this study is to investigate the effect and safety of two main analgesics (NSAIDs and opioids) in the treatment of patients diagnosed with acute renal colic, by a systematic review and meta-analysis.

NSAIDs exert their analgesic effect through inhibiting the release of prostaglandins in the body, then acting on the kidneys to increase the diuretic effect, which efficiently alleviates the pain of renal colic patients (Larkin et al., 1999). The creation of renal colic was attributed to prostacyclin and prostaglandin E2, which has been presented in previous studies (Fu et al., 2019). The above demonstration accounts for using NSAIDs in patients with acute renal colic. Be divergent with NSAIDs, however, opioids work by binding to opioid receptors in the brain of the patients and then stimulating the opioid systems that result in decreased pain within a short time.

A total of 18 studies (Hetherington and Philp, 1986; Thompson et al., 1989; Oosterlinck et al., 1990; Sandhu et al., 1994; Cordell et al., 1996; Larkin et al., 1999; Safdar et al., 2006; Bektas et al., 2009; Serinken et al., 2012; Azizkhani et al., 2013; Ay et al., 2014; Masoumi et al., 2014; Pathan et al., 2016a; Zamanian et al., 2016; Al et al., 2018; Sotoodehnia et al., 2019; Rezaei et al., 2020) were included in this systematic review and meta-analysis, involving 3,121 participants who used NSAIDs or opioids for releasing pain. The primary outcome in this study—changes in the VAS at 0–30 min, showed the result of the nonsignificant difference between the NSAID and opioid groups, which is consistent with the study of Khammissa (2020). The conclusion of this outcome is consistent with previous studies (Das and Teece, 2006), but there is one article that clearly indicates significant advantage in favor of NSAIDs in the treatment of acute renal colic (Steinberg and Chang, 2016). The subgroup analysis that was performed, in light of the different routes of administration (IV & IM administration) and NSAID type, with changes in the VAS at 0–30 min and the VAS at 60 min, aims to explore the resource of heterogeneity among the included studies about certain outcomes discussed in this systematic review and meta-analysis. Additionally, this study found interesting results that all of the secondary outcomes were statistically significant for patients with renal colic, between the NSAID and opioid groups. To be specific, regarding the outcome of the VAS over different time periods. There was no significant difference in the analgesic effect of NSAIDs and opioids on renal colic in each time period except 15 min. These results indicated that opioids that are more likely to be needed later in the rescue cause drug-related side effects in patients when compared with the NSAIDs group. Furthermore, using NSAIDs has no advantages on several outcomes of the VAS at 30, 45, 60 min and for the VAS at 0–30 min, but it seems to be a better choice when used at 15 min, when compared to opioids. A single article (Masoumi et al., 2014) might account for this different result on changes in the VAS at 0–30 min.

According to previous studies and this meta-analysis (Khammissa et al., 2020), the factors of analgesic effect of these two kinds of drugs are numerous and could be divided into two types, which involve the dose and routes of administration in patients, and the differences among individuals and environments (e.g., age, gender, and pain sensitivity).

On the one hand, a plethora of publications did not elucidate the method of administration, and at present, the evidence is inadequate to dictate the risk of this factor (Daoust et al., 2015). But, on the other hand, the beneficial effects of diclofenac sodium in the form of IM has been stated by Marthak et al. (1991); meanwhile, diclofenac sodium has been identified as a remarkably favored agent by assessing the overall effectiveness of the relevant drugs in the treatment of renal colic. Moreover, the RCT conducted by Pathan et al. (2016b) also found that IM NSAID drugs could be considered as the first-line treatment, as it provides the best analgesic effect for patients with renal colic. However, compared with opioids, both IV and IM administration of NSAIDs exhibited no better outcomes regarding change in the VAS at 0–30 min and 60 min. The reason for heterogeneity is possibly attributed to the different scales in assessing patients' pain and the antalgic agents those clinicians used for patients, based on the results of this meta-analysis.

Regarding the antalgic effects and side effects of both NSAIDs and opioids, whereas some publications persist that NSAIDs is superior to opioids in terms of side effects (e.g., hypotension, respiratory depression) (Esmailian and Keshavarz, 2014; Berthelot et al., 2015), there are a few studies that indicate there are similar effects between these two kinds of agents (Fu et al., 2019). Almost all included studies in this systematic review and meta-analysis illustrated adverse effects in their participants who were suffering acute renal colic and were using analgesics, but the divergent data about adverse effects, after analyzing the subgroup analysis, showed significant differences in favor of NSAIDs when compared with opioids in the treatment of renal colic patients. Similarly, a higher risk of side effects in patients when using opioids for treatment of renal colic, such as nausea and vomiting, was reported in many systematic reviews and meta-analyses, when compared with NSAIDs (Holdgate and Pollock, 2005). Besides, a recent study raised a regime that IV lidocaine could be a substitution for mitigating pain in a condition of intolerant adverse effects of NSAIDs or opioids (Loj et al., 2018). One note of caution, we considered there are possible reasons to explain the inconsistencies of side effects included in the studies reported, that is, the analgesic effect was not satisfactory due to the underdose of the antalgics and individual variations, because severe pain of acute renal colic can also cause nausea, vomiting, and hypertension (Holdgate and Pollock, 2005).

## Limitation

There are several potential limitations in this systematic review and meta-analysis. First, the potential influence, in terms of the dose of the agents used for acute renal colic patients, would probably trigger uncertain results on the efficacy and safety of NSAIDs and opioids in the treatment of renal colic, especially regarding the primary outcome changes in the VAS at 0–30 min. In addition, the reason for this difference is likely due to various drugs provided by the different hospitals. Further RCTs are urgently required to investigate whether different agents and dosage of drugs used for renal colic have diverse influence on patients while alleviating extreme pain.

## Conclusion

According to the results of this systematic review and meta-analysis. There is no significant difference between the NSAIDs and opioids in the treatment of renal colic in many outcomes (e.g., the VAS over different time periods except 15 min, using different injection methods at 30 and 60 min). However, patients can benefit from less side effects because clinicians use NSAIDs. Based on the subgroup analysis of NSAID and opioid types, ketorolac performs better than meperidine at 15 and 30 min, with acetaminophen and morphine performing equally, and ketorolac is better than fentanyl at 30 and 60 min, and acetaminophen is also better than morphine. Ketorolac also has a better performance than opioids in terms of drug side effects and whether rescue is required.

## Data Availability

The raw data supporting the conclusion of this article will be made available by the authors, without undue reservation.
