# Supplementary material for: Comparison of the Efficacy of Nonsteroidal Anti-Inflammatory Drugs and Opioids in the Treatment of Acute Renal Colic: A Systematic Review and Meta-Analysis
Source: Front Pharmacol. 2022 Jan 27;12:728908. doi: 10.3389/fphar.2021.728908 (PMC8828916; doi:10.3389/fphar.2021.728908)
Supplement: Supplementary file 1 [file DataSheet1.DOCX]

**Legends of Supplemental Materials**

**Supplemental Method 1** Detailed search strategy.

**Supplemental Figure 1** Quality assessment of included studies.

**Supplemental Figure 2** Funnel plot of changes in pain scores (VAS) at 0 to 30 minutes.

**Supplemental Figure 3** Funnel plot of dizziness caused by the medications

**Supplemental Figure 4** Funnel plot of pain scores at 60 minutes

**Supplemental Figure 5** Funnel plot of need for rescue analgesia

**Supplemental Figure 6** Funnel plot of pain scores (VAS) over different time periods

**Supplemental Figure 7** Funnel plot of routes of adminstration for changes in VAS at 0 to 30 minutes

**Supplemental Figure 8.** Funnel plot of the frequency of side-effects caused by the medications

**Supplemental Figure 9.** Funnel plot of vomiting caused by the medications

**Supplemental Method 1** Detailed search strategy.

**Ovid MEDLINE(R) Epub Ahead of Print, In-Process & Other Non-Indexed Citations, Ovid MEDLINE(R) Daily, Ovid MEDLINE and Versions(R) <1946 to February 01, 2021>**

#1 exp Urinary Bladder Calculi/ or exp Urinary Calculi/ or exp Kidney Calculi/ or exp Ureteral Calculi/ or exp Urolithiasis/ or exp Nephrolithiasis/ or exp Renal Colic/ or exp Ureteral Diseases/ or exp Ureteral Obstruction/ or exp Kidney Diseases

#2 ((Urin* or renal or kidney or ureter* or bladder) adj3 (stone* or calcul* or colic* or lith* or obstruct* or occlusi*)).mp.

#3 ((Kidney or ureter*) adj2 diseas*).mp.

#4 (Urolith* or nephrolith*).mp.

#5 or/1-4/

#6 exp Anti-Inflammatory Agents, Non-Steroidal/ or exp Cyclooxygenase Inhibitors/ or exp Cyclooxygenase 2 Inhibitors

#7 ((Nonsteroidal adj2 antiinflammatory) or (Non-steroidal adj2 antiinflammatory) or (Nonsteroidal adj2 antiinflammatory) or (Non-steroidal adj2 antiinflammatory)).mp.

#8 exp diclofenac/ or exp ketorolac/ or exp apazone/ or exp aspirin/ or exp ibuprofen/ or exp ketoprofen/ or exp Salicylates/ or exp etodolac/ or exp naproxen/ or exp indomethacin/ or exp piroxicam/ or exp celecoxib/ or exp fenoprofen/ or exp sulindac/ or exp tolmetin/ or exp mesalazine/ or exp aminosalicylic acid

#9 NSAID*.mp.

#10 (Diclofenac or adiflam or agile or diclonac or dicol or diclonat* or feloran or voltarol or Voltaren or Cataflam or Voltaren-XR or Zipsor).mp.

#11 (Aceclofenac or Hifenac or Cincofen or Nacsiv or Acenac).mp.

#12 (Ketorolac or toradol or torolac or kealc or kenalfin or ketlac).mp.

#13 (Apazon* or Azapropazon* or cinnopropazon*).mp.

#14 (Aspirin* or acetylsal* or dispril or easprin* or salicylic*).mp.

#15 (Ibuprofen or brufen or nuprin or rufen or salprofen or dolgit or salprofen or advil* or motrin or nurofen or actiprofen or addaprin or aktren or anadin or bugesic or ibuprox).mp.

#16 (ketoprofen or orudis or oruvail or ketoflam or oruvail or fastum or ketonal or ketodol or knavon or actron or ketoprofeno).mp.

#17 (Dexketoprofen or keral or enantyum or ketesgel or dolmen).mp.

#18 (Naproxen or naprosyn or naprosin or proxen or synflex or Aleve or Anaprox or Apronax or Naprelan).mp.

#19 (etodolac or ramodar or ultradol or etova or dualgan or etodin or etopan or flancox or proxym or etodine or dolarit).mp.

#20 (Indomethacin* or indocid or indocin or indomet or indometacin or metindol or osmosin).mp.

#21 (Piroxicam or feldene or dolocare or dolonex or ketolin).mp.

#22 (Meloxicam or mobic or vivlodex).mp.

#23 (Tenoxicam or mobiflex).mp.

#24 (celecoxib or celebrex or celebra).mp.

#25 (rofecoxib or vioxx or ceoxx or ceeoxx).mp.

#26 (valdecoxib or bextra).mp.

#27 (Nimesulid* or aulin or mesulid or nimalox or sulid* or sintalgin or nimsid* or nise or nimulid).mp.

#28 (Meclofenamic or meclofenamat* or meclomen).mp.

#29 (fenoprofen or fenopron).mp.

#30 (oxaprozin or oxaprozinum or daypro or dayrun or duraprox).mp.

#31 (sulindac or cinoril or imbaral).mp.

#32 (tolmetin or tolectin).mp.

#33 (flurbiprofen* or sulindac* or mesalazin* or sulfasalazin* or flufenamic* or tolmetin* or fenoprofen* or diflunisal* or niflumic* or ketorolac or trometamol* or parecoxib* or teriflunomid* or benoxaprofen* or suprofen* or fenbufen* or mebron* or mepirizole* or mepyrizole* or methopyrimazole* or Epirizolum* or Polihexanid* or Dalex* or Miton* or epirizol* or clonixin* or tolemetin* or nabumeton*).mp.

#34 or/6-33

#35 exp Analgesics, opioid/ or exp alkaloids, opiate/ or exp narcotics

#36 opioid*.mp.

#37 exp hydrocodone/ or exp dextropropoxyphene/ or exp fentanyl/ or exp meperidine/ or exp methadone/ or exp Morphine/ or exp Morphine Derivatives/ or exp oxymorphone/ or exp pentazocine/ or exp tramadol

#38 exp alfentanil/ or alfentanil*.mp.

#39 exp codeine/ or codein*.mp.

#40 (hydrocodon* or vicodin* or norco or lortab or zohydro).mp.

#41 exp oxycodone/ or oxycodon*.mp.

#42 (dextropropoxyphen* or darvon or darvocet or digesic or capadex).mp.

#43 exp dihydromorphine/ or dihydromorphin*.mp.

#44 (fentanyl or actiq or duragesic or fentora or sublimaz* or fenta).mp.

#45 (meperidin* or demerol or pethidin*).mp.

#46 (methadon* or dolophin* or methadose or amidon* or symoron or physephton* or heptadon).mp.

#47 (morphin* or oramorph or morphia or duramorph or contin or mscontin or sevredol or zomorphzomo).mp.

#48 (oxymorphon* or numorphan or opana or morphon).mp.

#49 (pentazocin* or fortal or sosegon or talwin or fortwin or talacen).mp.

#50 (tramadol or ultram).mp.

#51 or/35-50

#52 34 or 51

#53 randomized controlled trial.pt.

#54 controlled clinical trial.pt.

#55 (random$ or placebo$ or single blind$ or double blind$ or triple blind$).ti,ab.

#56 (retraction of publication or retracted publication).pt.

#57 or/53-56

#58 (animals not humans).sh.

#59 ((comment or editorial or meta-analysis or practiceguideline or review or letter or journal correspondence) not "randomized controlled trial").pt.

#60 (random sampl$ or random digit$ or random effect$ or random survey or random regression).ti,ab. not "randomized controlled trial".pt.

#61 or/58-60

#62 57 not 61

#63 5 and 52 and 62/ (852)

**Ovid EMbase <** **1974 to 2021 February 01 >**

#1 exp Urinary Bladder Calculi/ or exp Urinary Calculi/ or exp Kidney Calculi/ or exp Ureteral Calculi/ or exp Urolithiasis/ or exp Nephrolithiasis/ or exp Renal Colic/ or exp Ureteral Diseases/ or exp Ureteral Obstruction/ or exp Kidney Diseases

#2 ((Urin* or renal or kidney or ureter* or bladder) adj3 (stone* or calcul* or colic* or lith* or obstruct* or occlusi*)).mp.

#3 ((Kidney or ureter*) adj2 diseas*).mp.

#4 (Urolith* or nephrolith*).mp.

#5 or/1-4/

#6 exp Anti-Inflammatory Agents, Non-Steroidal/ or exp Cyclooxygenase Inhibitors/ or exp Cyclooxygenase 2 Inhibitors

#7 ((Nonsteroidal adj2 antiinflammatory) or (Non-steroidal adj2 antiinflammatory) or (Nonsteroidal adj2 antiinflammatory) or (Non-steroidal adj2 antiinflammatory)).mp.

#8 exp diclofenac/ or exp ketorolac/ or exp apazone/ or exp aspirin/ or exp ibuprofen/ or exp ketoprofen/ or exp Salicylates/ or exp etodolac/ or exp naproxen/ or exp indomethacin/ or exp piroxicam/ or exp celecoxib/ or exp fenoprofen/ or exp sulindac/ or exp tolmetin/ or exp mesalazine/ or exp aminosalicylic acid

#9 NSAID*.mp.

#10 (Diclofenac or adiflam or agile or diclonac or dicol or diclonat* or feloran or voltarol or Voltaren or Cataflam or Voltaren-XR or Zipsor).mp.

#11 (Aceclofenac or Hifenac or Cincofen or Nacsiv or Acenac).mp.

#12 (Ketorolac or toradol or torolac or kealc or kenalfin or ketlac).mp.

#13 (Apazon* or Azapropazon* or cinnopropazon*).mp.

#14 (Aspirin* or acetylsal* or dispril or easprin* or salicylic*).mp.

#15 (Ibuprofen or brufen or nuprin or rufen or salprofen or dolgit or salprofen or advil* or motrin or nurofen or actiprofen or addaprin or aktren or anadin or bugesic or ibuprox).mp.

#16 (ketoprofen or orudis or oruvail or ketoflam or oruvail or fastum or ketonal or ketodol or knavon or actron or ketoprofeno).mp.

#17 (Dexketoprofen or keral or enantyum or ketesgel or dolmen).mp.

#18 (Naproxen or naprosyn or naprosin or proxen or synflex or Aleve or Anaprox or Apronax or Naprelan).mp.

#19 (etodolac or ramodar or ultradol or etova or dualgan or etodin or etopan or flancox or proxym or etodine or dolarit).mp.

#20 (Indomethacin* or indocid or indocin or indomet or indometacin or metindol or osmosin).mp. / (41784)

#21 (Piroxicam or feldene or dolocare or dolonex or ketolin).mp.

#22 (Meloxicam or mobic or vivlodex).mp.

#23 (Tenoxicam or mobiflex).mp.

#24 (celecoxib or celebrex or celebra).mp.

#25 (rofecoxib or vioxx or ceoxx or ceeoxx).mp.

#26 (valdecoxib or bextra).mp.

#27 (Nimesulid* or aulin or mesulid or nimalox or sulid* or sintalgin or nimsid* or nise or nimulid).mp.

#28 (Meclofenamic or meclofenamat* or meclomen).mp.

#29 (fenoprofen or fenopron).mp.

#30 (oxaprozin or oxaprozinum or daypro or dayrun or duraprox).mp.

#31 (sulindac or cinoril or imbaral).mp.

#32 (tolmetin or tolectin).mp.

#33 (flurbiprofen* or sulindac* or mesalazin* or sulfasalazin* or flufenamic* or tolmetin* or fenoprofen* or diflunisal* or niflumic* or ketorolac or trometamol* or parecoxib* or teriflunomid* or benoxaprofen* or suprofen* or fenbufen* or mebron* or mepirizole* or mepyrizole* or methopyrimazole* or Epirizolum* or Polihexanid* or Dalex* or Miton* or epirizol* or clonixin* or tolemetin* or nabumeton*).mp.

#34 or/6-33

#35 exp Analgesics, opioid/ or exp alkaloids, opiate/ or exp narcotics

#36 opioid*.mp.

#37 exp hydrocodone/ or exp dextropropoxyphene/ or exp fentanyl/ or exp meperidine/ or exp methadone/ or exp Morphine/ or exp Morphine Derivatives/ or exp oxymorphone/ or exp pentazocine/ or exp tramadol

#38 exp alfentanil/ or alfentanil*.mp.

#39 exp codeine/ or codein*.mp.

#40 (hydrocodon* or vicodin* or norco or lortab or zohydro).mp.

#41 exp oxycodone/ or oxycodon*.mp.

#42 (dextropropoxyphen* or darvon or darvocet or digesic or capadex).mp.

#43 exp dihydromorphine/ or dihydromorphin*.mp.

#44 (fentanyl or actiq or duragesic or fentora or sublimaz* or fenta).mp.

#45 (meperidin* or demerol or pethidin*).mp.

#46 (methadon* or dolophin* or methadose or amidon* or symoron or physephton* or heptadon).mp.

#47 (morphin* or oramorph or morphia or duramorph or contin or mscontin or sevredol or zomorphzomo).mp.

#48 (oxymorphon* or numorphan or opana or morphon).mp.

#49 (pentazocin* or fortal or sosegon or talwin or fortwin or talacen).mp.

#50 (tramadol or ultram).mp.

#51 or/35-50

#52 34 or 51

#53 randomized controlled trial.pt.

#54 controlled clinical trial.pt.

#55 (random$ or placebo$ or single blind$ or double blind$ or triple blind$).ti,ab.

#56 (retraction of publication or retracted publication).pt.

#57 or/53-56

#58 (animals not humans).sh.

#59 ((comment or editorial or meta-analysis or practiceguideline or review or letter or journal correspondence) not "randomized controlled trial").pt.

#60 (random sampl$ or random digit$ or random effect$ or random survey or random regression).ti,ab. not "randomized controlled trial".pt.

#61 or/58-60

#62 57 not 61

#63 5 and 52 and 62/ (4278)

**Cochrane Central Register of Controlled Trials** **in Cochrane Reviews <Issue 2 of 12, February 2021>**

#1 ((kidney pain) or (kidney colic) or (bladder stone) or (ureter stone) or (urolithiasis) or (nephrolithiasis) or (ureter obstruction)):ti,ab,kw

#2 ((NSAID) or (nonsteroid antiinflammatory agent) or (cyclooxygenase 2 inhibitor) or (indometacin) or (piroxicam) or (acetylsalicylic acid) or (celecoxib) or (diclofenac) or (ibuprofen) or (naproxen) or (azapropazone) or (acetylsalicylic acid) or (ketoprofen) or (salicylic acid) or (ketorolac) or (ketoprofen) or (salicylic acid derivative) or (etodolac) or (fenoprofen) or (sulindac) or (tolmetin) or (mesalazine) or (aminosalicylic acid)):ti,ab,kw

#3 ((Opioids) or (opiate) or (narcotic analgesic agent) or (opiate agonist) or (hydrocodone) or (dextropropoxyphene) or (fentanyl) or (pethidine) or (methadone) or (oxymorphone) or (pentazocine) or (tramadol)):ti,ab,kw

#4 #2 or #3

#6 #1 and #4 / (935)


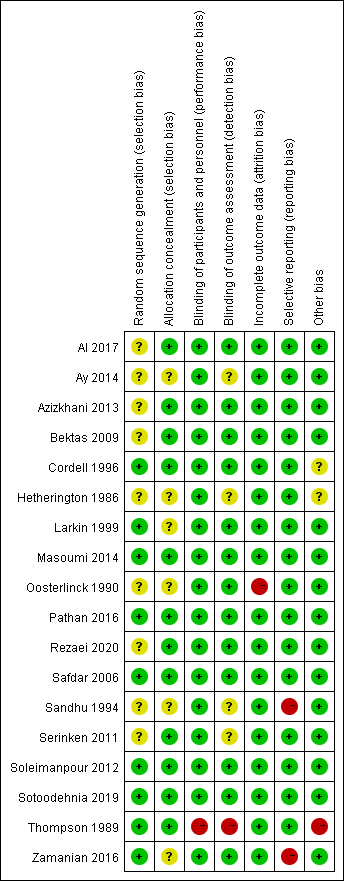


**Supplemental Figure 1** Quality assessment of included studies.


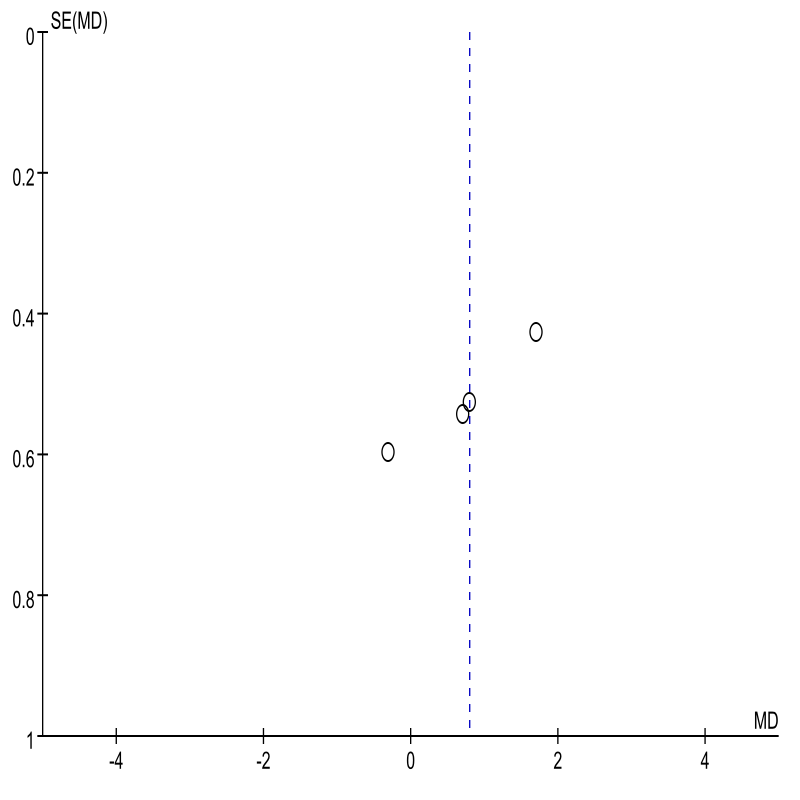


**Supplemental Figure 2** Funnel plot of changes in pain scores (VAS) at 0 to 30 minutes.


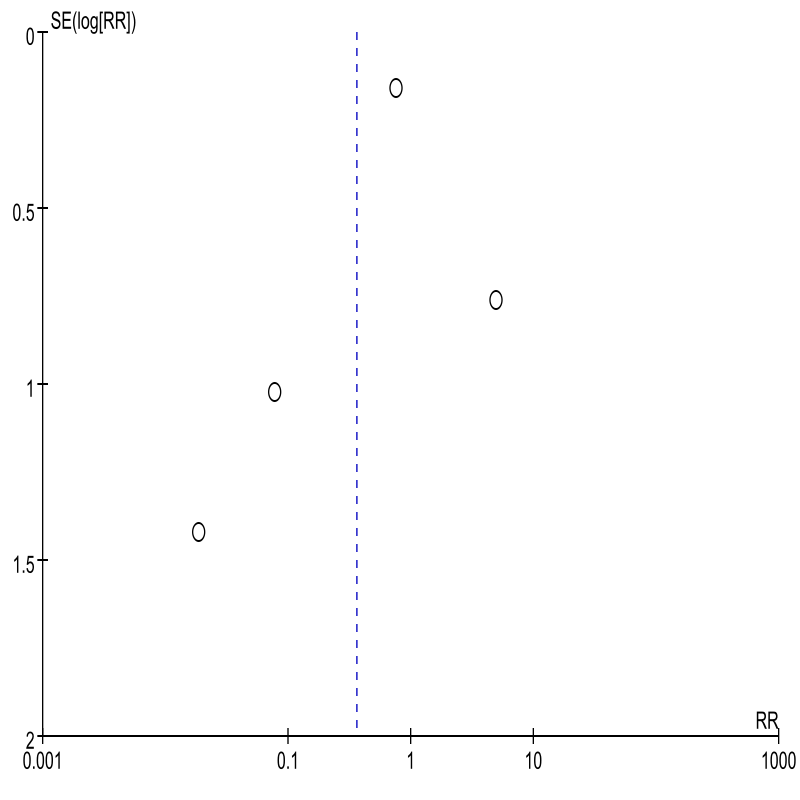


**Supplemental Figure 3** Funnel plot of dizziness caused by the medications


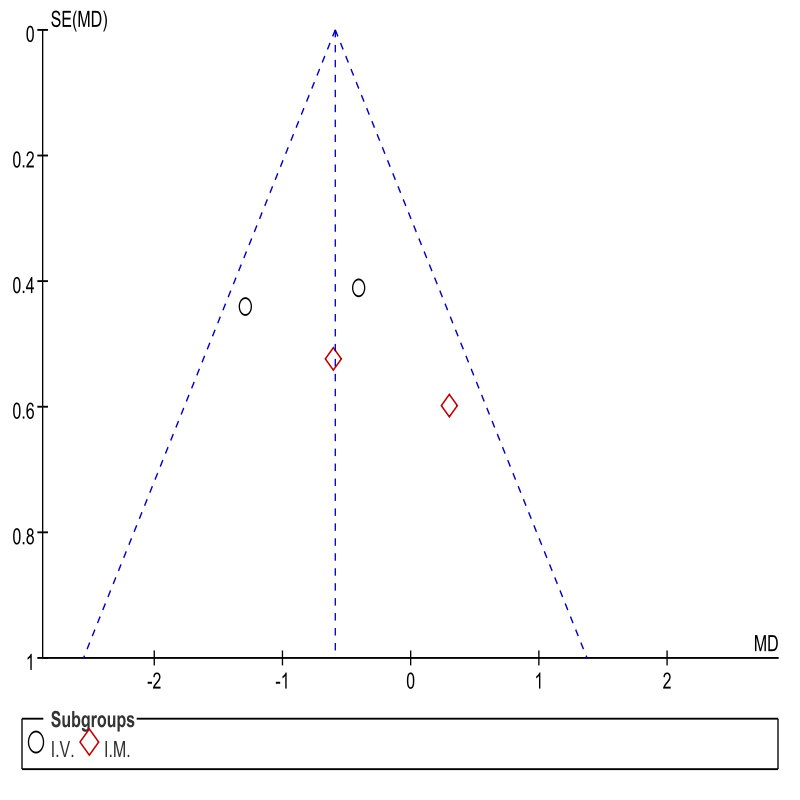


**Supplemental Figure 4** Funnel plot of pain scores at 60 minutes


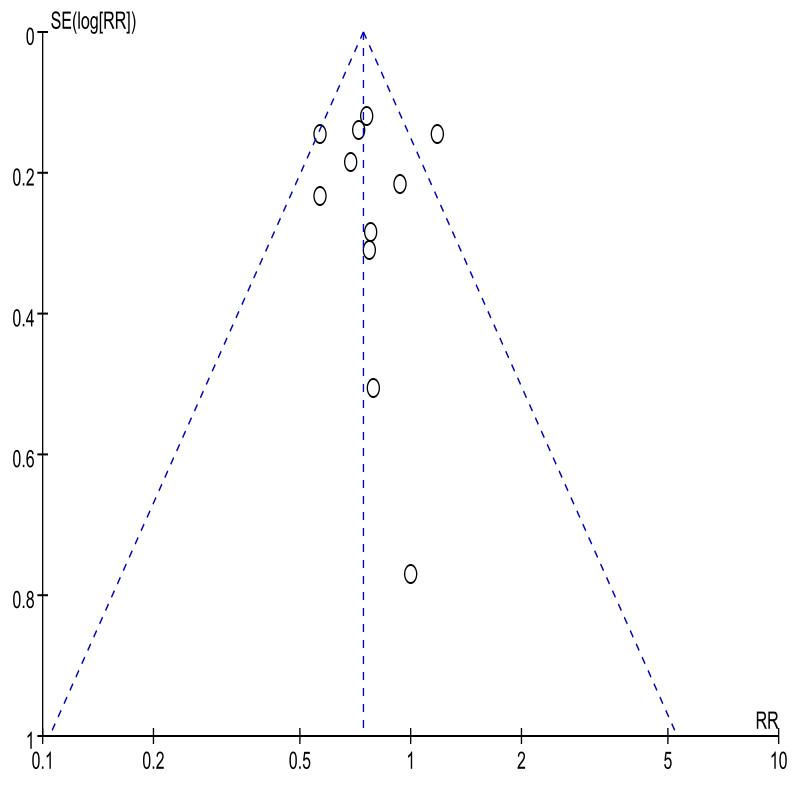


**Supplemental Figure 5** Funnel plot of need for rescue analgesia


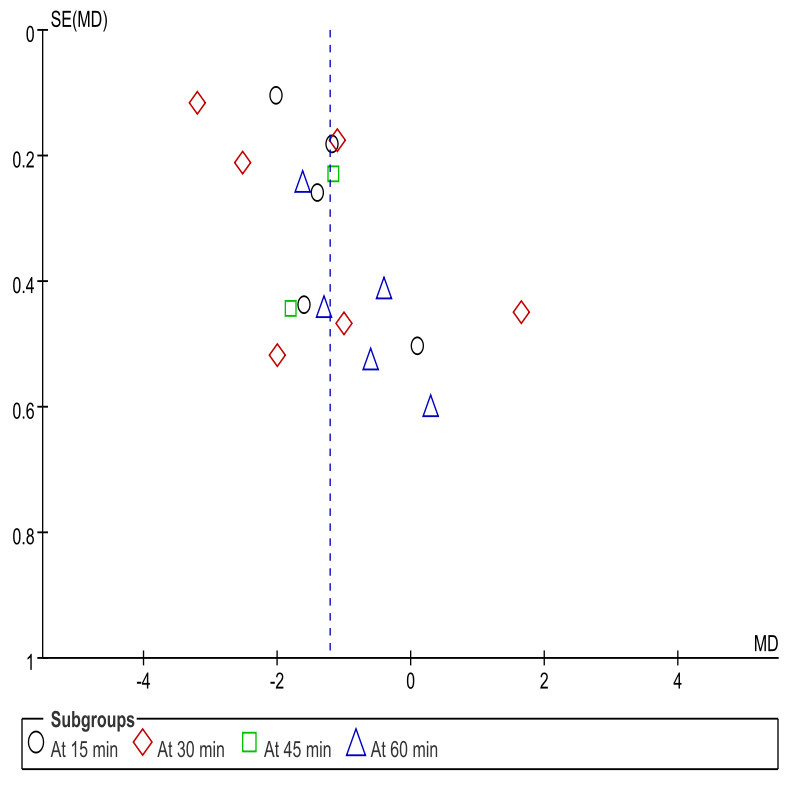


**Supplemental Figure 6** Funnel plot of pain scores (VAS) over different time periods


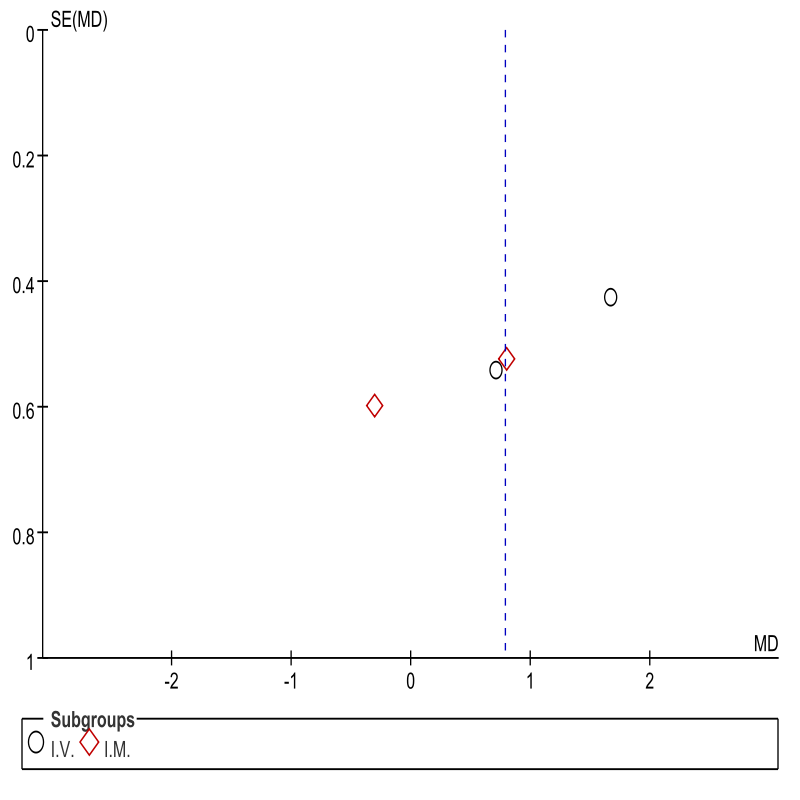


**Supplemental Figure 7** Funnel plot of routes of adminstration for changes in VAS at 0 to 30 minutes


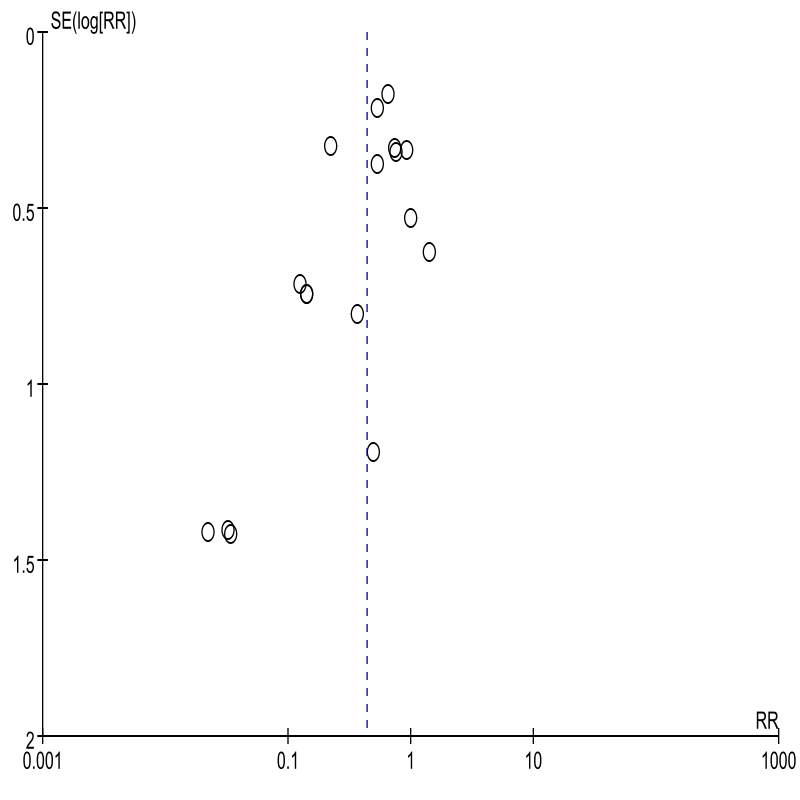


**Supplemental Figure 8** Funnel plot of the frequency of side-effects caused by the medications


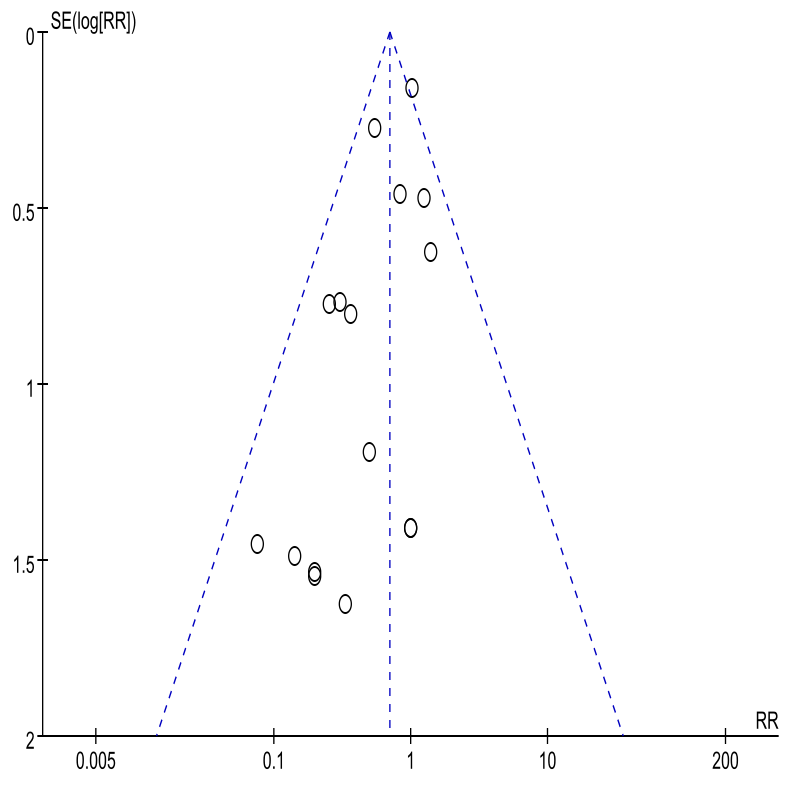
**Supplemental Figure 9** Funnel plot of vomiting caused by the medications
